# Stem–Loop Structures in Iron-Regulated mRNAs of *Giardia duodenalis*

**DOI:** 10.3390/ijerph20043556

**Published:** 2023-02-17

**Authors:** Laura Y. Plata-Guzmán, Rossana Arroyo, Nidia León-Sicairos, Adrián Canizález-Román, Héctor S. López-Moreno, Jeanett Chávez-Ontiveros, José A. Garzón-Tiznado, Claudia León-Sicairos

**Affiliations:** 1 Programa Regional del Noroeste para el Posgrado en Biotecnología de la Facultad de Ciencias Químico-Biológicas, Universidad Autónoma de Sinaloa, Av. de las Américas y Josefa Ortíz (Cd. Universitaria), Culiacán 80030, Mexico; 2 Departamento de Infectómica y Patogénesis Molecular, Centro de Investigación y de Estudios Avanzados del IPN (CINVESTAV-IPN), Av. IPN No. 2508, Colonia San Pedro Zacatenco, Mexico City 07360, Mexico; 3 CIASaP Facultad de Medicina, Universidad Autónoma de Sinaloa, Cedros y Sauces Frac. Fresnos, Culiacán 80246, Mexico; 4 Departamento de Investigación, Hospital Pediátrico de Sinaloa, Boulevard Constitución S/N, Col. Jorge Almada, Culiacán 80200, Mexico

**Keywords:** *Giardia duodenalis*, foodborne disease, giardiasis etiology, anemia comorbidities, iron regulation, IREs

## Abstract

*Giardia duodenalis* is a significant cause of waterborne and foodborne infections, day-care center outbreaks, and traveler’s diarrhea worldwide. In protozoa such as *Trichomonas vaginalis* and *Entamoeba histolytica,* iron affects the growth, pathogenicity mechanisms, and expression of virulence genes. One of the proposed iron regulatory mechanisms is at the post-transcriptional level through an IRE/IRP-like (iron responsive element/iron regulatory protein) system. Recently, the expression of many putative giardial virulence factors in the free-iron levels has been reported in subsequent RNAseq experiments; however, the iron regulatory mechanism remains unknown. Thus, this work aimed to determine the effects of iron on the growth, gene expression, and presence of IRE-like structures in *G. duodenalis*. First, the parasite’s growth kinetics at different iron concentrations were studied, and the cell viability was determined. It was observed that the parasite can adapt to an iron range from 7.7 to 500 µM; however, in conditions without iron, it is unable to survive in the culture medium. Additionally, the iron modulation of three genes was determined by RT-PCR assays. The results suggested that *Actin*, *glucosamine-6-phosphate deaminase*, and *cytochrome b5* mRNA were down-regulated by iron. To investigate the presence of IRE-like structures, in silico analyses were performed for different mRNAs from the Giardia genome database. The Zuker mfold v2.4 web server and theoretical analysis were used to predict the secondary structures of the 91 mRNAs analyzed. Interestingly, the iron-induced downregulation of the genes analyzed corresponds to the location of the stem–loop structures found in their UTR regions. In conclusion, iron modulates the growth and expression of specific genes, likely due to the presence of IRE-like structures in *G. duodenalis* mRNAs.

## 1. Introduction

*Giardia duodenalis* is the etiologic agent of giardiasis, one of the most common causes of waterborne and foodborne infections, which is characterized by extensive diarrhea [1]. Approximately 200 million people are infected with *G. duodenalis* annually, mainly in Latin America, Asia, and Africa [2,3]. *G. duodenalis* is of biological interest since this parasite occupies a key point of the evolutionary transition between prokaryotes and eukaryotes. It has similar properties to prokaryotic cells, such as a lack of mitochondria and peroxisomes as well as bacterial-type metabolic enzymes. However, it belongs to the first-known divergent eukaryotic lineage [4,5,6]. Moreover, in *G. duodenalis,* the great need for iron is reflected in the anemia it causes, especially in children with giardiasis [7]. After treatment with metronidazole, an increase in hemoglobin in the cured children’s blood is observed [8,9]. The likelihood of giardiasis occurring is higher in malnourished individuals [7,10]. Thus, iron could play a key role in Giardia virulence [8,9,10,11]. Despite this, there are almost no studies regarding the effect of iron on the growth, gene expression, or regulatory mechanisms in *Giardia*. Recently, Peirasmaki et al. (2020) [12] reported on the expression of many putative giardial virulence factors in free-iron levels detected by RNAseq experiments [12]; however, the iron regulatory mechanism remains unknown.

Iron is an essential element for all living organisms. Because both a deficiency in and surplus of iron are harmful, the absorption, concentration, and redox status should be carefully regulated [13,14,15,16]. The post-transcriptional regulation carried out by the IRE/IRP system is the most studied iron regulatory mechanism in mammalian cells. This mechanism consists of cytoplasmic iron regulatory proteins (IRPs) that interact with iron-responsive elements (IREs), which are conserved stem–loop structures found in untranslated regions (UTRs) of the mRNA-encoding proteins related to iron homeostasis [13,14,15,16]. In protozoans such as *T. vaginalis* and *E. histolytica*, iron affects growth and pathogenicity mechanisms [17,18,19,20,21], as well as the expression of some mRNA-encoding virulence proteins [18,22,23,24,25,26]. In *T. vaginalis,* the presence of IRE-like structures in *tvcp4* and *tvcp12* mRNAs that are differentially regulated by iron at the post-transcriptional level [23,27,28], as well as the presence of a 135 kDa α-actinin that behaves similarly to an RNA-binding protein under iron-restricted conditions and binds to these structures, have been reported [29].

However, in *G. duodenalis*, the presence of IRE-like structures in some of its mRNAs has not been reported. Infection with *G. duodenalis* is closely related to malnutrition in children, reflecting the clinical importance of the need for iron in the case of being infected with this parasite. Despite this, studies related to the effect of iron on the growth or pathogenicity of the parasite or the modulation of the gene expression under different iron concentrations have not been reported regarding this parasite. Thus, this work aimed to determine the effect of iron on parasite growth and gene expression and to search for the presence of IRE-like structures through an in silico analysis of *Giardia* mRNAs. The results suggest that iron modulates the growth and expression of specific genes, likely due to the presence of IRE-like structures in *G. duodenalis* mRNAs. This study on the effect of iron on the regulatory system in *G. duodenalis* aims to help us understand the evolutionary process of iron regulation and the biology of the parasite.

## 2. Materials and Methods

### 2.1. Parasite Cultures

The WB isolate of *G. duodenalis* was axenically grown in TYI-S-33 medium enriched with 10% fetal bovine serum (Gibco, Thermofisher, Lifetehcnologies Corporation, Grans Island, NY, USA), 1 g/L bile (Sigma-Aldrich, St. Louis, MO, USA), pH 7.0, and 50 μg/mL gentamicin and filtered through 0.45 μm membrane (Merck Millipore Ltd., Tullagreen, Carrigtwohill, Co. Cork, IRL). The trophozoites of *Giardia* were harvested at 4 °C and centrifuged at 432 sp. gr. for 5 min [30]. Then, the supernatant was decanted; the pellet was resuspended in 1.5 mL of bovine fetal serum, homogenized and resuspended in 13.5 mL of a new culture medium, and incubated at 37 °C for 24 h [31]. To obtain high iron condition (HI) parasite cultures, 200, 300, and 500 μM of ferrous ammonium citrate (Sigma-Aldrich) were added to the medium, or Chelex-100 resin (BioRad, Hercules, CA, USA) was added to obtain an iron-restricted (IR) parasite culture (7.7 μM final concentration) [26,30,31]. The parasites were treated with 0.5 g/100 mL Chelex-100 and sterilized after the removal of the resin by filtration. The final concentration of iron in the cultures was measured by a spectrophotometer (Thermo Scientific, Waltham, MA, USA).

### 2.2. Iron Effect on the G. duodenalis Growth

To analyze the effect of iron on the *G. duodenalis* growth, the culture medium TYI-S-33 was obtained under normal iron conditions (NI, 100 μM), as described above [30,31]. To perform the growth kinetics, 5.0 × 10^6^ trophozoites grown under NI conditions were inoculated in different iron conditions (0, HI, and IR) and incubated up to 120 h at 37 °C. Then, the cultures in the different iron conditions were centrifuged at 432 sp. gr. at 4 °C for 5 min. The pellet was washed with 1× PBS and homogenized, and the parasites were counted in the Neubauer chamber. The viability of *G. duodenalis* was evaluated by the trypan blue method in an inverted microscope (Leica Microsystems, Wetzlar, Germany).

### 2.3. Reverse Transcriptase PCR (RT-PCR) Assays

The total RNA from 2 × 10^7^ parasites grown in IR (7.7 μM) and HI (300 μM) conditions was obtained using the TRIzol reagent (Sigma-Aldrich). For RT-PCR assays, RNA treated with DNase I to remove contaminating DNA was reverse-transcribed using AMV (Avian Myeloblastosis Virus, MI, USA) reverse transcriptase and an oligo (dT) primer (Thermo Scientific). Primers for amplifying *Actin*, *cytochrome b5*, and *glucosamine-6-phosphate deaminase* mRNA fragments were designed using Oligo 7.2 Software (Molecular Biology Insights, Inc., Cascade, CO, USA), according to the published sequence in the database of the *G. duodenalis* genome [30,31] (ID GL50803_40817, ID GL50803_9089, and GL50803_114625, respectively). The primers were used to amplify a 296 bp fragment of the *Actin* gene (5′-GAGAAAAGATTTGCCAGA-3′ forward and 5′-CTCTTTTATATCACGCAC-3′ reverse), a 419 bp fragment of the *cytochrome b5* gene (5′-AATTTTAATGAGTGAACA-3′ forward and 5′-ATTGTTTACTTTCTCGCC-3′ reverse), and a 350 bp fragment of the *glucosamine-6-phosphate deaminase* gene (5′-AGCAAGGTCAATATAAAG-3′ forward and 5′-CCTCACACTTGCTCTCAC-3′ reverse). A 507 bp amplicon of the *G. duodenalis β-tubulin* mRNA (5′-AGATCGTCCACATCCAGGCA-3′ forward and 5′-CTTCGGGGACGGGACGACGG-3′ reverse) was used as an internal control. The PCR conditions for *Actin* were 28 cycles of 94 °C for 45 s, 47.7 °C for 30 s, and 72 °C for 1 min, followed by a final extension step of a single cycle at 72 °C for 5 min. The PCR conditions for *cytochrome b5* were 28 cycles of 94 °C for 45 s, 50.6 °C for 30 s, and 72 °C for 1 min, followed by a final extension step of a single cycle at 72 °C for 5 min. The PCR conditions for *glucosamine-6-phosphate deaminase* were 26 cycles of 94 °C for 45 s, 47.8 °C for 30 s, and 72 °C for 1 min, followed by a final extension step of a single cycle at 72 °C for 5 min. The amplicons were analyzed on a 1% agarose gel with gel red staining (Biotium, Fremont, CA, USA). A densitometric analysis using a GelDoc (Bio-Rad) and Quantity One-4.6.3 1-D Analysis Software (Biolinx labsystems, Mahape, Navi Mumbai, India) was performed. The values for *β-tubulin* expression were used to normalize the results of the *Actin*, *cytochrome b5*, and *glucosamine 6 phosphate deaminase* mRNAs expression under both iron conditions tested [26].

### 2.4. Prediction of Stem–Loop Structures in G. duodenalis mRNAs

To obtain the sequences that encode the virulence, cytoskeleton, ribosomal, and metabolic proteins, we used the database of the *G. duodenalis* genome (http://dgiardiadb.org/giardiadb/, accessed on 18 July 2021) [32,33]. The ends of the sequences were delineated in the following manner. The 5′-UTR region was described according to the Knodler and Elmendorf reports [34,35]. These authors propose A/T-rich regions as a transcription initiator. An A in the middle of the A/T-rich sequence could be the best transcription start site [36]. In the 3′-UTR region, the polyA AGTPuAA consensus signal was searched [37], which is slightly degenerated in positions 1, 2, and 3, as well as the ATGGAA signal located 9–13 bp upstream from where polyadenylation is carried out [38]. In addition, the reported motifs, as putative polyadenylation signals (AGUGAA, GUGAAC, of which AGUGAA is the most common motif identified in *G. duodenlis*), were searched according to Franzen et al., 2013 [39]. Once the sequences were delineated, the secondary structures were analyzed by the Zuker mfold program (http://www.unafold.org/mfold/applications/rna-folding-form.php, accessed on 20 July 2021) [40].

## 3. Results

### 3.1. Iron Affects the Growth and Viability of G. duodenalis

**Figure 1:**
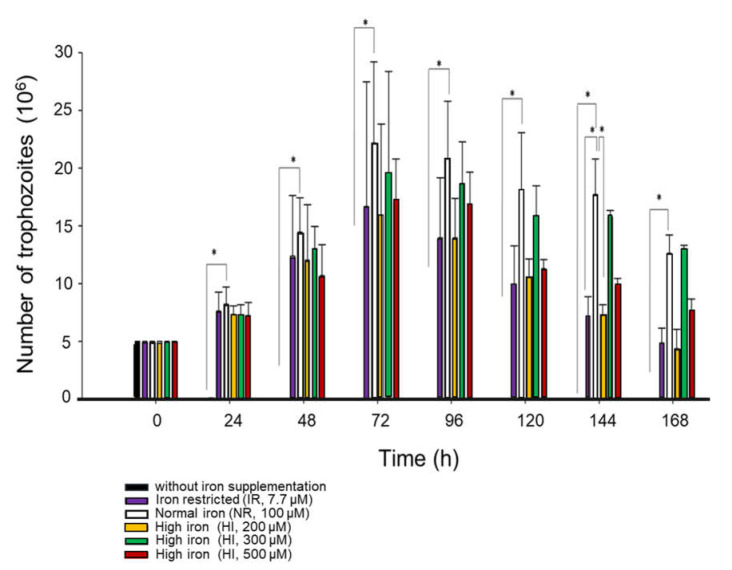
Iron’s effect on the growth and viability of *G. duodenalis*. The culture was initiated by inoculating 5 × 10^6^ trophozoites into 15 mL of culture media. As indicated, the *Giardia* growth was measured when grown in the medium supplemented with different iron concentrations. Experiments were performed in triplicate. Statistical significance is indicated with an asterisk.

To elucidate the effect of iron on the growth of *G. duodenalis*, the growth kinetics were determined at different iron culture concentrations (0, 7.7, 100, 200, 300, and 500 μM) for up to 120 h at 37 °C; the number, viability, and morphology of the parasites were evaluated using an inverted microscope (Leica). After 24 h (t_1_), the number of parasites was 7.6 × 10^6^ trophozoites under IR (7.7 μM), 8.2 × 10^6^ trophozoites in NI (100 μM), 7.2 × 10^6^, 7.1 × 10^6^, and 7.2 × 10^6^ trophozoites under HI (200, 300, and 500 μM of iron), and 7.5 × 10^4^ trophozoites in the control culture without iron. When the incubation time was extended (t_2_ = 48 h), the number of parasites increased, meaning that there was a greater number of parasites under IR conditions than there was under HI conditions. The parasite number reached its maximum value after being cultured for 72 h: 16.6 × 10^6^ trophozoites under IR (7.7 μM); 22.1 × 10^6^ trophozoites in NI (100 μM); and 15.9 × 10^6^, 19.5 × 10^6^, and 17.2 × 10^6^ trophozoites under HI (200, 300, and 500 μM of iron). However, after 96 h and 120 h, the number of parasites decreased in all iron conditions, possibly suggesting that the parasite can adapt to different iron conditions. Thus, in the absence of iron, a drastic reduction in parasite growth was observed in the 7.7 to 100 μM range (Figure 1), suggesting that iron is an important element for the growth of *G. duodenalis* trophozoites. However, the parasite can adapt to different iron concentrations from 7.7 up to 500 μM (Figure 1).

### 3.2. Actin-Related Protein, Cytochrome b5, and Glucosamine-6-Phosphate Deaminase Are Differentially Modulated by Iron

**Figure 2:**
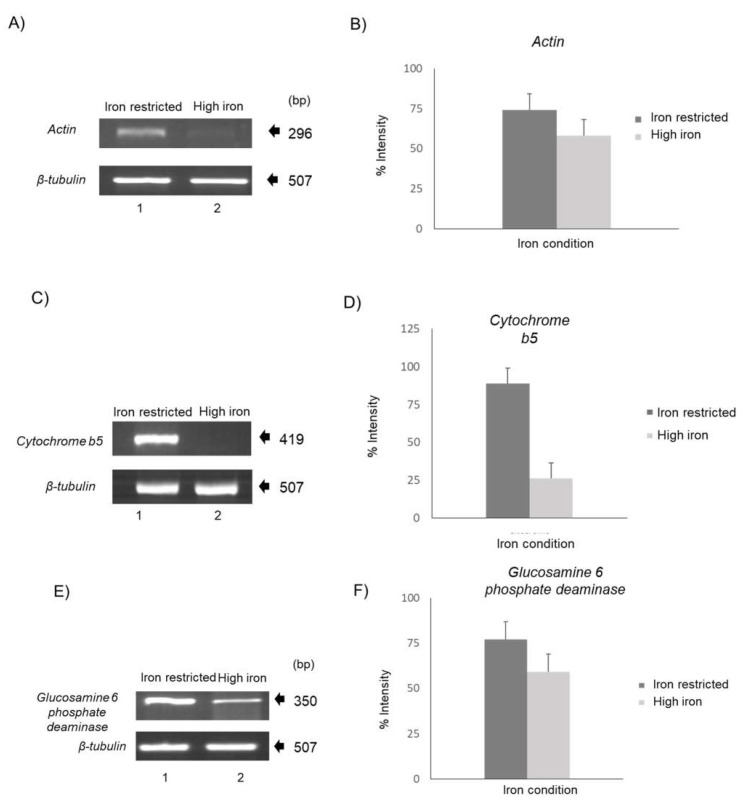
Iron conditions differentially modulate *Actin*, *Cytochrome b5*, and *Glucosamine 6 phosphate deaminase*. (**A**) Semiquantitative RT-PCR assays with *Actin* gene-specific primers using cDNA from parasites grown under iron-restricted (7.7 mM; lane 1) and high-iron (300 mM; lane 2) conditions. The *β-tubulin* gene was used as an internal control. The sizes of the amplicons are given in bp. (**B**) Densitometric analysis using a GelDoc (BioRad) with Quantity One-4.6.3 1-D Analysis Software. (**C**) Semiquantitative RT-PCR assays with specific primers for the *Cytochrome b5* gene using cDNA from parasites grown under iron-restricted (7.7 mM; lane 1) and high-iron (300 mM; lane 2) conditions. The *β-tubulin* gene was used as an internal control. The sizes of the amplicons are given in bp. (**D**) Densitometric analysis using a GelDoc (BioRad) with Quantity One-4.6.3 1-D Analysis Software. (**E**) Semiquantitative RT-PCR assays with specific primers for *Glucosamine-6-phosphate deaminase* using cDNA from parasites grown under iron-restricted (7.7 mM; lane 1) and high-iron (300 mM; lane 2) conditions. The *β-tubulin* gene was used as an internal control. The sizes of the amplicons are given in bp. (**F**) Densitometric analysis was performed using a GelDoc (BioRad) with Quantity One-4.6.3 1-D Analysis Software. Values of *β-tubulin* expression were used to normalize the results of *Actin*, *Cytochrome b5*, and *Glucosamine-6-phosphate deaminase* mRNA expression under both iron conditions. Experiments were performed in triplicate.

To investigate whether iron affects *G. duodenalis* virulence genes, as has been reported in *E. histolytica* and *T. vaginalis* [18,21,23,26], we performed semiquantitative RT-PCR assays of *Actin*, *cytochrome b5*, and *glucosamine-6-phosphate deaminase* to demonstrate iron regulation. All three genes were negatively regulated by iron, showing overexpression under IR conditions (Figure 2A–F). The expression of these three genes was similar to that of the *E. histolytica Ehhmbp26* gene [24], whose RNA sequence formed two IRE-like hairpins at both UTRs. However, the IRE-like structure at the 3′ UTR appeared to be more stable [26]. Thus, we hypothesize that these giardial iron-downregulated genes have stem–loop structures in the mRNA of their UTRs.

### 3.3. Presence of Stem–Loop Structures in G. duodenalis mRNAs

To search for the mRNA-stem–loop elements involved in iron regulation, we performed a bioinformatic analysis of the *Giardia* genome sequence, selecting various virulence, ribosomal, cytoskeleton, and metabolic enzyme mRNAs. First, the untranslated regions were identified and delineated, as has been reported [30,31,32,33,34,35,36,37,38,39,40]. *Giardia* genes have a short 6 nt sequence at the 5′ UTR mRNA that can act as a ribosomal anchoring site for protein synthesis [41]. The promoter regions of this parasite appear to be very compact, and the intergenic regions are very short (sometimes less than 250 bp). This parasite contains short (1–6 nt) UTR regions. However, these regions have the elements required for transcriptional regulation—specifically, an A/T-rich region. The initiator element of this parasite consists of 8–11 nt (position −30 in ATTAAAA) [34,35,36,37,38,39,42] with its variants. An A in the middle of the A/T-rich sequence could be the best transcription initiation site [34]. Holberton and Marshall [42] suggested that the transcription initiation site sequence is composed of nine bases (AATTAAAAA) and is associated with other sequences, such as the CAATTT promoter.

In addition, a potential polyadenylation signal 20–30 nt downstream from the stop codon (AGTPuAApy) has been reported [38]. In the 3′ UTR signal, the consensus polyA AGTPuAA [33] has been found and is slightly degenerated in positions one, two, and three; an ATGGAA signal located 9–13 nt upstream from the polyadenylation site has also been detected [36]. In addition, Franzen et al. (2013) reported putative polyadenylation signals as motifs of AGUGAA and GUGAAC, of which AGUGAA is the most common motif identified in *G. duodenalis* [39]. The tetranucleotide sequence UAAA has been shared by several motifs as part of the canonical eukaryotic polyadenylation signal (AAUAAA), suggesting that *G. duodenalis* polyadenylation signals may be more similar to the canonic eukaryotic AAUAAA than had been previously recognized. Moreover, in that study, a subset of genes was missing any clear motif, suggesting that a strict polyadenylation may not be required for proper 3′ end formation, which occurs in higher eukaryotes (Franzen et al., 2013) [39]. According to previous reports, in this work, the 5′ and 3′ UTRs were delineated (Tables S1–S3, available in the online Supplementary Materials). Thereby, the delineated regions of the 5′ and 3′ UTRs were used to search for the formation of a stem–loop structure using Zuker mfold v2.4 webserver [40]. In this analysis, 91 sequences formed hairpin IRE-like secondary structures. Interestingly, 38 of the 91 mRNAs showed stem–loop structures at both the 5′ and 3′ UTRs in all of the analyzed groups. However, we observed increased stability for one of the two structures formed in one of the UTRs for each mRNA. This stability is based on dG and the presence of reported motifs (a reduction in dG (negative dG) is a necessary condition for the spontaneity of the structure’s formation) [40].

**Figure 3:**
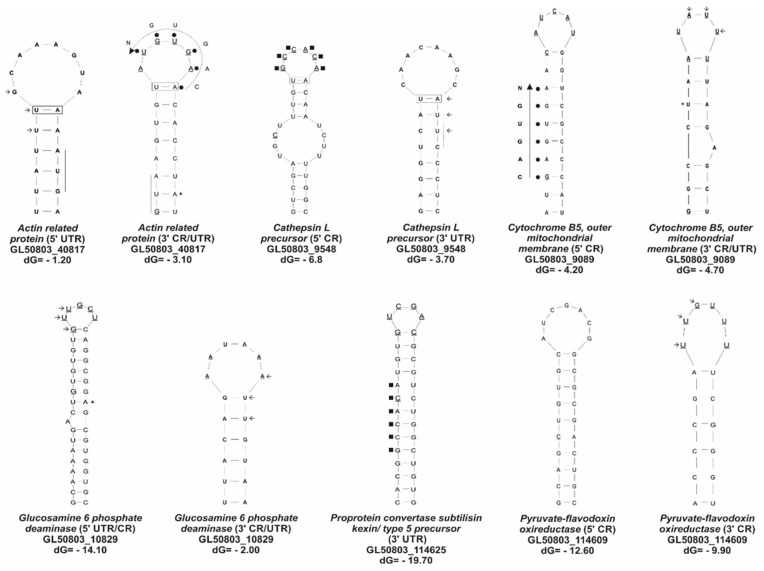
Prediction of stem–loop structures of *G. duodenalis* mRNAs coding for virulence, metabolism, and cytoskeleton proteins. The six nucleotides that form the apical loop of the canonical IREs are underlined. Nucleotides U-A/A-U in the rectangle indicate conserved nucleotides in the upper stem. Filled black circles indicate nucleotides similar to the CAGUGN consensus human motif, and a large or curved arrow indicates the 5′ to 3′ or 3′ to 5′ orientations of the sequence. The GUU/UUG protozoa-specific motif is indicated in small arrows. Asterisk: residue U conserved on canonical IREs. Small triangles show the residue A conserved in the protozoa-specific motif. Small squares indicate a similar motif to *tvcp4*-IRE reported. dG, Gibbs free energy or free enthalpy; in this case, a reduction in dG (negative dG) is a necessary condition for the spontaneity of the structure formation.

As expected, the three genes downregulated by iron, *actin-related protein* (GL50803_40817), *cytochrome b5* (GL50803_9089), and *glucosamine-6-phosphate deaminase* (GL50803_10839), formed two IRE-like structures at both UTRs, including the coding region, as was reported for the *tvcp4*-IRE, *Actin*-IRE, and *Ehhmbp26*-IRE of *E. histolytica* [23,26]. In the loop, the 3′ end of *Actin*-IRE possesses an atypical sequence compared to the human consensus sequence [39], and U-A residues were conserved in the upper stem [42]. Additionally, the 3′ ends of *cytochrome b5*-IRE and *glucosamine-6-phosphate deaminase*-IRE have an unpaired nucleotide in the stem and the AUU motif in the loop, which is similar to the GUU-specific motif of protozoan parasites [27] (Figure 3). Only ten sequences shared similarity with the conserved human loop (CAGUGN) [42], corresponding to an Actin-related protein (at the 3′ coding region (CR) and the UTR) and the outer mitochondrial membrane of cytochrome b5 (GL50803_9089, at the 5′ CR), as well as to some sequences annotated as ribosomal proteins (Supplementary Figure S2). Most of the hairpin structures contain the A-U conserved nucleotides in the upper stem, and some stem–loops have residues at positions 1–5 that form a pseudotriloop, as has been reported in higher eukaryotes [42,43,44,45]. Moreover, some sequences contain a motif similar to the *tvcp4*-IRE (GGCACA), such as the precursor to cathepsin L (GL50803_9548) and the IRE-like structure at the 5′ coding region [23]. Interestingly, the GUU loop motif, which was reported as a protozoa-specific IRE-like motif [27], was present in all of the analyzed groups (Figure 3 and Figures S1–S4).

Interestingly, two new motifs were also found in the giardial IRE-like structures: the CUU/AUU sequences that are present in 28 of the 91 mRNAs analyzed (Figure 3 and Figures S1–S4), suggesting the presence of new motifs for this parasite or a new variant of the GUU loop motif, reported as a protozoa-specific motif [27]. However, some structures do not exhibit any of these motifs in the loop; these sequences were found in the stem’s structure. Piccinelli and Samuelsson (2007) [46] conducted a bioinformatic analysis of mRNA sequences for more than 100 novel sequences—for instance, for ferritin, the transferrin receptor, mitochondrial aconitase, ferroportin, and DMT1 from different species. These sequences formed IRE-type structures; in the loop, they presented the CAGUGN sequence, and in some IREs, they presented a bulge with the UGC/C nucleotides in the stem with an unpaired cytosine. Despite the absence of the canonical CAGUGN sequences in the loop, in some IRE-like structures from *G. duodenalis* determined through our bioinformatic analysis, the UGC/C bulge was observed, such as in *Cathepsin L precursor* (5′CR) (GL50803_9548), *Dipeptidyl peptidase III* (5′UTR/CR) (GL50803_3822), and *Ribosomal protein L37* (3′CR/UTR) (GL50803_14171). In addition, in the stem of the sequences of the *Cathepsin B precursor* (5′CR) (GL50803_14019) and the *Cathepsin B precursor* (5′CR) (GL50803_16779), there is the CGC loop, which is similar to the UGC reported by Piccinelli and Samuelsson (2007) [46]. These data suggest that there are some UGC/C-like IREs in Giardia, which could represent an ancestral version of the ferritin IRE. It is possible that *G. duodenalis* contains different types of IRE-like structures in the primordia that evolved until they reached the canonical IRE sequence reported for the metazoans. In addition, an analysis in the SIREs web server was performed, and the results are in the Supplementary Materials (Table S5 and attached materials). Although not all of the stem–loop structures analyzed by Zucker showed the formation of IREs in the SIREs web server, some of them, such as *Pyruvate-flavodoxin oxidoreductase* GL50803_114609, *Cathepsin L precursor* GL50803_3169, *Cathepsin L-like protease* GL50803_3099, *Hypothetical protein* GL50803_113303, *Ribosomal protein L7a* GL50803_17244, *Ribosomal protein L12* GL50803_14938, and *Actin-related protein* GL50803_8726, showed the formation of typical metazoan IREs (Figure 3, Table S5, attached materials). Thus, a giardial IRP-like or RNA-binding protein may recognize the sequence in the loop or the stem’s structure. The data in this work show in silico evidence of cis-elements, such as IRE-like structures in the mRNA of *G. duodenalis* coding for various groups of proteins, including those involved in virulence.

## 4. Discussion

Iron is an essential cation for the growth of most microorganisms, including protozoan pathogens [47]. Some of them have developed hemoglobin-binding proteins, reductases, and transporters [47,48]. Iron is obtained from the direct lysis of host cells by protozoa. However, the acquisition of iron is regulated to avoid the toxicity induced by the formation of free radicals. For example, in *E. histolytica* and *T. vaginalis,* iron regulates many of the proteins involved in cytoadherence and cytotoxicity, other virulence factors, and the molecules involved in growth and the basal metabolism [17,18,19,20,21,22,23,24,25,26]. The recent observation of therapies against protozoan pathogens based on the combination of compounds is currently a very promising direction in the drug discovery field, and iron chelation is under study [47]. *Giardia’s* great need for iron is reflected in the anemia, which it causes, especially in children with giardiasis [9], where, after treatment with metronidazole, there is an increase in hemoglobin in the blood [8,9,10]. However, there are few studies related to the effect of iron on the growth and virulence of this parasite. Recently, Peirasmaki et al. (2020) [12] reported on how iron caused a downregulation of a broad repertoire of genes in *Giardia* trophozoites during an interaction with IECs in vitro [12]; in this work, the analyzed transcripts were downregulated using a different metal ion chelator in the medium. In this work, additional assays were included to demonstrate how iron affects the growth and regulation of the gene expression in *G. duodenalis*. To determine the effect of iron, growth kinetics were carried out at different iron concentrations. We observed a drastic reduction in the number of *Giardia* trophozoites without iron supplementation, which indicates that this parasite requires this metal in order to grow; this also occurs in other pathogens [18,21,26,28,47,48,49]. It would be interesting to study iron chelation as a chemotherapeutic alternative in *Giardia* growth under different iron conditions. In addition, it would be interesting to study the synergistic effect of lactoferrin, a molecule of the immune system, and iron as a therapy against giardiasis, since the effect of only lactoferrin as an anti-giardial has been demonstrated [50]. This possible synergistic effect is currently under study in our lab.

Moreover, our data suggest that *G. duodenalis* possibly uses different molecules to survive, depending on the iron conditions. This metal is fundamental for the duplication of the parasite, as suggested by the growth kinetics, where a marked reduction in the culture viability was observed when using chelating iron and not a cation source. Instead, by using a range of 7.7 to 500 μM of iron in the culture, we were able to observe that the parasite can adapt in a similar manner to what occurs in amoeba reports since, in a similar study carried out in *E. histolytica* [21], the growth of the amebae did not differ among the low-iron (21.4 μM), normal iron (71.4 μM), and high-iron (285.6 μM) groups. In all three groups, the viability of the amebae was higher than 80% after 72 h of culture. It is possible that iron differentially modulates virulence in *G. duodenalis*, as has been reported in *E. histolytica* [18,21,24,25,26] and *T. vaginalis* [17,19,20,22,23]. In this study, a marked decrease in parasite viability was observed over long periods of time in media under IR conditions, reflecting the parasite’s need for iron to survive.

In addition, the negative iron modulation correlates with the location of the IRE-like structures in the 3′ UTR of *Actin*, *glucosamine-6-phosphate deaminase*, and *cytochrome b5* mRNA using RT-PCR assays. Actin is necessary for the cell morphology, growth, and encystment of this parasite [51,52]. Another enzyme that participates in the encystment process of *Giardia* is *glucosamine-6-phosphate deaminase* [53]; it also has a specific function in the life cycle and metabolism of *G. duodenalis* [53,54]. The encystation process in *G. duodenalis* is triggered by environmental changes, such as cholesterol deprivation, the bile content, and pH value; however, the specific molecular mechanisms regulating cell differentiation remain unclear [55]. The results from this work suggest that iron conditions could be another environmental change that can participate in this complex mechanism. On the other hand, the results that suggest that *cytochrome b5* is negatively regulated by iron show the fundamental role of this protein in low-iron or iron-restricted conditions in obtaining the cation and surviving the hostile microenvironment [46,55,56]. Similar to the human transferrin receptor, cytochrome contains a stem–loop structure in its 3′ UTR region; however, although it is a single structure and is not canonical, it could show an IRE-like function.

The analysis of these IRE-like structures showed that these mRNAs can form IRE-like structures at both UTRs. The parasite likely uses the IRE of the extreme that has a more negative dG in combination with the typical elements of the IREs which have already been reported [26,27,45]. These IRE-like structures showed that the GUU protozoa-specific motif reinforces what was previously reported for protozoan IREs [27], and some of them showed the formation of typical metazoan IREs, as determined by Zuker and the SIREs web program (Figure 3 and Figures S1–S4) [27,45].

Due to the presence of an IRE-like structure at the 3′ UTR of the mRNA and the negative iron regulation, the genes analyzed in this study could be regulated at the post-transcriptional level by an IRE/IRP system, which occurs with the transferrin receptor, which contains five IRE structures in the 3′ UTR region. Thus, under IR conditions, an IRP-like protein can be attached to the stem–loop structures, providing stability to the mRNA and carrying out its translation. The fact that *G. duodenalis* regulates several genes depending on the iron conditions may represent one of the most primitive IRE/IRP-like regulatory mechanisms in eukaryotes in its early stages of evolution. In addition, the data in this work show in silico evidence of cis-elements such as IRE-like structures in *G. duodenalis* mRNAs coding for various groups of proteins. On the other hand, we searched orthologous IRP-like proteins by conducting an in silico analysis of the proteome of *G. duodenalis*, using the human IRPs sequences as probes. The results showed that the GL50803_14790 sequence had an identity of 32% to IRP-1, which was annotated as a hypothetical protein in the giardial genome. However, no nucleic acid-binding domains were found by the in silico analysis. It is possible that this protein with a low homology and without nucleic acid-binding domains could not be the IRP-like protein in Giardia. Therefore, the identification of a possible IRP-like protein was carried out using Western blot assays, which suggests that there was mainly a recognition of a protein band in IR conditions by the anti-human IRP-1 antibody (Supplementary Figure S5); it is also possible that another protein of a different molecular size has an IRP-like function in giardia, perhaps a multifunctional protein, as occurs in *T. vaginalis* [28,29]. Thus, additional experiments are necessary to determine the mRNA stability and to identify the *G. duodenalis* RNA-binding protein which could interact with the IRE-like hairpin structures reported in this study. This mechanism could be used to regulate different genes using iron. In this case, it could be involved in the virulence to adapt to fluctuating changes in iron concentrations to those that are beyond those only involved during iron metabolism, as has been reported in higher eukaryotes, which represents a specialization of this type of iron homeostasis in this regulation system. Our results suggest that *G. duodenalis*, which is evolutionarily close to *E. histolytica* and *T. vaginalis,* could also have an IRE/IRP-like mechanism. Finally, the presence of this mechanism in *G. duodenalis* remains under study by our research group.

## Data Availability

Not applicable.

## Supplementary Materials

The following are available online at https://www.mdpi.com/article/10.3390/ijerph20043556/s1, Figure S1: Prediction of stem–loop structures of *G. duodenalis* mRNAs coding for virulence proteins; Figure S2. Prediction of stem–loop structures of *G. duodenalis* mRNAs coding for ribosomal proteins; Figure S3: Prediction of stem-loop structures of *G. duodenalis* mRNAs coding for cytoskeleton proteins; Figure S4. Prediction of stem–loop structures of *G. duodenalis* mRNAs coding for metabolic proteins. Figure S5. Immunodetection of a cytoplasmic protein in *G. duodenalis* by an anti-human IRP-1 antibody; Figure S6: Attached material showing the sequences of some mRNAs presented in our study and analyzed by SIRES web server; Table S1. Ends of sequences coding virulence proteins. Table S2. Ends of sequences coding ribosomal proteins. Table S3. Ends of sequences coding cytoskeleton proteins. Table S4. Ends of sequences coding metabolic proteins. Table S5. IRE-like structures predicted by the SIREs web server of some mRNA from *G. duodenalis*. Attached material showing SIREs analysis.
