# Supplementary material for: Stem–Loop Structures in Iron-Regulated mRNAs of Giardia duodenalis
Source: Int J Environ Res Public Health. 2023 Feb 17;20(4):3556. doi: 10.3390/ijerph20043556 (PMC9966554; doi:10.3390/ijerph20043556)
Supplement: Supplementary file 1 [file ijerph-20-03556-s001.zip › ijerph-2098791-supplementary.pdf]

Supplementary Material

Communication

Stem–Loop Structures in Iron-Regulated mRNAs of *Giardia duodenalis*

Laura Y. Plata-Guzmán [1], Rossana Arroyo [2], Nidia León-Sicairos [3,4], Adrián Canizález-Román [3], Héctor S. López-Moreno [1], Jeanett Chávez-Ontiveros [1], José A. Garzón-Tiznado [1] and Claudia León-Sicairos [1,*]

[1] Programa Regional del Noroeste para el Posgrado en Biotecnología de la Facultad de Ciencias Químico-Biológicas, Universidad Autónoma de Sinaloa, Av. de las Américas y Josefa Ortíz (Cd. Universitaria), Culiacán 80030, Mexico
[2] Departamento de Infectómica y Patogénesis Molecular, Centro de Investigación y de Estudios Avanzados del IPN (CINVESTAV-IPN), Av. IPN No. 2508, Colonia San Pedro Zacatenco, Mexico City 07360, Mexico
[3] CIASaP Facultad de Medicina, Universidad Autónoma de Sinaloa, Cedros y Sauces Frac. Fresnos, Culiacán 80246, Mexico
[4] Departamento de Investigación, Hospital Pediátrico de Sinaloa, Boulevard Constitución S/N, Col. Jorge Almada, Culiacán 80200, Mexico
* Correspondence: claudialeonsicairos@uas.edu.mx

Table S1. Ends of sequences coding virulence proteins

Annotation or common name	Gene ID	Region	Sequence delineated
Cathepsin B precursor	GL50803_14019	5´CR	cuuuuccucc*aauuu[u]aaa*AUGaagcucuuucuccuc**gccgcugcagcuuucucugcgcccgc**ccugacggucuccgagc
Cathepsin B precursor	GL50803_14019	3´UTR	ugagUAA**gcaaugaauacucu*UGU***_AAAC_ggaaga[u]cuca
Cathepsin B precursor	GL50803_16160	5´UTR	guauucuagcagg*uauuuau[a]guuu*cucacca**guuAUGgaugcauugauugagccaaac**augcuuuuagcuaaagucuuu
Cathepsin B precursor	GL50803_16160	3´UTR	acuaugcagacguagagUGAaucc**gcuuuuguccucaacugugucugguggucgguuaagcuaaaaagu**uacagcgggcauggaccuugaucuccgg*AUGAACG*ccgaa[g]agauuaugca
Cathepsin B precursor	GL50803_16779	5´CR	aagcuuacgg*aauu[u]uaa*AUGaagcucuuucuccuc**gccgcugcagcuuucucugcgcccgc**ccugacggucuccgagcu
Cathepsin B precursor	GL50803_16779	3´CR/UTR	cauugaagagcaaguaau**agguggguucuuugagaacUAAcuggugaauccauuu**uau*AGUAAAU*caugg[c]caggagggu
Cathepsin B precursor	GL50803_10217	3´UTR	gac**AUGcgccugucuucauacauagcua**cca**uugcccuaaugguag**a*AGUAAAC*guuuuu[c]ugguccaugcauuaaaaa
Cathepsin B precursor	GL50803_15564	5´CR	cuagcauggc*uuuauuua[u]uu*gcucaucuuauggucagacggcaccAU**Gcgcgcgggaauccucugguggcgc**ugucggu
Cathepsin B precursor	GL50803_16468	3´UTR	uggcagguagcagugaagUAAacg**uccaaaggcuuuuuggg**aaaa*AGUCAUG*cccggg[u]guaucuuugcugcacggccgc
Cathepsin B precursor	GL50803_17516	5´CR	cuuauacaggaauuugcuccuuugaaggaacagag*aaauccu[u]uaaa*AU**Gaggcuucugauccuucugauugcauc**ccua
Cathepsin B-like cysteine proteinase 3	GL50803_114165	5´CR	ugcuccgggaacggcc*acggaa[a]uaaa*AU**Gaugguacuucuaccauu**cuugcuauugcuauauggcacacuuggcagaga
Cathepsin B-like cysteine proteinase 3	GL50803_114165	3´UTR	acuuucacgguacuagaugacacucauUG**Acacacucugaugaacuagau*GGU***_AAAU_cuaucc[u]aagcaaagauguuguc
Cathepsin L precursor	GL50803_3169	5´CR	aauaguuacaaacugcaa*ucuaagac[a]ucu*c**acAUGuuagcgcuauugcucuuuauugu**cugcuguguuucccaugaucc
Cathepsin L precursor	GL50803_3169	3´UTR	ugcugcaggaagUAGgugcggaugacuucua**acuauuagugaauaguagu**gaau*AGUAAUA*gacuag[u]ggacaacuuagg

Cathepsin L precursor	GL50803_16380	3´CR/UTR	uacgugaugguu**gagUAAauugcccggguuggugaaugugcuc**uggccuug*GUCAAAA*aaug[u]ccuugccaagucauaaa
Cathepsin L precursor	GL50803_14983	3´CR/UTR	agcuacgcgguaguuaa**caaaagaaaaagcUAAaguaaauucugug**gacgcugucuaauu*AGUGCAA*ucagg[u]gacuugg
Cathepsin L precursor	GL50803_17607	3´UTR	uugggUGAcuauaua**ugguuacuuaaggagcgcuauagcccccucaaacuggcca**accuuugc*AUGGUAC*ucugc[u]gugu
Cathepsin L-like protease	GL50803_3099	5´CR	guaaauaacgaaaau*aauau[u]uaaaa*gaaccaua**AUGuauacuuuguugguguucau**acugucgaauccucugcauaucc
Cathepsin L-like protease	GL50803_137680	5´UTR/CR	guguaaaaccugaagg*acuuuu[a]ua****aaa*****guagugAUGugguuguccuauuuu**cuggugaucuuuuugcuugaacucuuug
Cysteine protease	GL50803_112831	5´UTR/CR	cggcuaaccuacccgcggccc*aa[a]a****aagga*****ccggAUGuugcugcuuacuuugucuuu**ccucugcgcggcaucggccgagc
Cysteine protease	GL50803_112831	3´CR/UTR	uacaucuuccacagcccgcucaaccugaaccuuccgcacaa**gggcgugUAGcgccuagcg*****CCC****GAAC*uccccg[u]cguaga
Cysteine protease	GL50803_113656	5´UTR/CR	cggcuaaccuacccgcggccc*aa[a]a****aagga*****ccggAUGuugcugcuuaccuugucuuu**ccucugcgcggcgucggccgagc
Cysteine protease	GL50803_113656	3´CR/UTR	uacaucuuccacagcccccucaaccugaaccuuccgcacaa**gggcgugUAGcgccuagcg*****CCC****GAAC*uccucg[u]cguaga
Cysteine protease	GL50803_114773	5´UTR/CR	cggcuaaccuacccgcagc*ccaa[a]a****aa*****ggaccggAUGuugcugcuuaccuugucuuu**ccucugcgcggcaucggccgagc
Cysteine protease	GL50803_114773	3´CR/UTR	ccucaaccugaaccuuccgcacaa**gggcgugUAGcgccu**agcg*CCCGAAC*ucccc*[g]*ucguagaggguggucagguagaug
Cysteine protease	GL50803_114915	5´UTR/CR	cggcuaaccuauccgcggccc*aa[a]a****aaggau*****cggAUGuugcugcuuacuuugucuuu**ccucugcgcggcgucggccgagc
Cysteine protease	GL50803_114915	3´CR/UTR	uacaucuuccacagcccgcucaaccugaaccuuccgcacaa**gggcgugUAGcgccuagcg*****CCC****GAAC*uccccg[u]cguaga

Dipeptidyl-peptidase III	GL50803_3822	5´UTR/CR	agggggg*aauauaa[a]uu****u*****ccAUGcugccugagaaguauauuugcccgacagaugga**cccguuucuaacuugaagccaucg
Dipeptidyl-peptidase III	GL50803_3822	3´UTR	ggaaaUAAaug**cgacguuguaaguug**auuguaauaacugcuuagaucacaucuauucuaggggga*GGUAAAG*augg[u]augg
Dipeptidyl-peptidase I precursor	GL50803_14566	5´CR	aacaauaagaagccggcc*agaa[a]aaaau*cAUGcuuuuca**ucuuggcgcuccuggga**cucuuauuaucagauacuccagca
Dipeptidyl-peptidase I precursor	GL50803_28651	3´UTR	cugaccaugcauuaUA**Gaugcacugaguguuuaacugagugcuuauc**gc*CAUAAAG*acucaccuau[u]cucuacaauaaua
Dipeptidyl-peptidase III	GL50803_33689	5´CR	gcuagcuuuuacugugaauuauuc*aauuu[u]ucua*AUGccuaac**gaaugcccaucuggagcuuuc**acggucaaccugaagc
Dipeptidyl-peptidase III	GL50803_33689	3´UTR	guaacaacauccuuguuuccucugcuaa**acUGAcuauacagu**gucuauauucucga*GCUUAAU*cagcaac[u]gcuugugaau
Encystation-specific protease	GL50803_22553	3´CR/UTR	caggaggagagugaggagcuaagca**ugcuuucaaauccaUGA*AGUA***AAU*gauuccacaag[u]acaauuacaaaacagccau
Hypothetical protein	GL50803_6369	5´UTR/CR	ccucuagag*auga[u]gaaa*cgagagaugaga**cucauAUGgacuacuggauugcucguaacucuuggggag**aagagugggga
Hypothetical protein	GL50803_113303	3´UTR	uucacgguauuggaugacacucauUG**Acacacucugaugaacuagau*GGU***AAAU*cuaucc[u]aagcaaagauguugucggu
Sentrin specific protease	GL50803_16438	3´UTR	aagaucuggacgagcucu**gcUAGgccuaugggucuggu**gucuaaguuguguu*UUGAAAA*cccuac[u]cugcccguguuaggg
Variant-Specific Surface Protein 150 (VSP-150)	GL50803_135831	5´CR	ugugcucaagg*uacu[u]cuuaua*cAUGaauggcuguuacaagg**uuggucaagaaccag**guagucauaugugcaagacagc
Variant-Specific Surface Protein 150 (VSP-150)	GL50803_135831	3´UTR	cuggugguucauauguagagggaagg**caUGAcucaggu*AGUG***AAU*gcua[u]acaaugugugauaaggguaauagagggu
Variant-Specific Surface Protein 5 (VSP-5)	GL50803_113797	5´UTR	ccccgggcuuugcguuggaagcgcca[c]ccagc**aggucggcggccu**aAUGuucggcagauuuuugcucgcgaucgucaucc
Variant-Specific Surface Protein 5 (VSP-5)	GL50803_113797	3´UTR	ugcuggugguuccucugcagggggaaggcgUAGaug**uacuuagau*AGUA***AAC*cguca[u]cgaugggucugcucggugucug

Variant-Specific Surface Protein 277 (VSP-277)	GL50803_14307	5′UTR/CR	caggggccugcgacgccaucgugguc[g]gcaac**gacggcagcAUGcucuc**uuacuguucaaaguguguuggcgccggcuau
Variant-Specific Surface Protein 277 (VSP-277)	GL50803_14307	3′UTR	ccucugcugguggu ucgugugua gagggaaggcgUG**Acuuaggu*AGU***<u>*GAAU*</u>gcug[u]gcagugugugauagugggugauau

Delineation of the untranslated regions of certain sequences encoding for ribosomal proteins in *G. duodenalis*. At the 5′end: CAATTT element: shading. T/A-rich region: italics. Transcription start site: brackets. Start codon: Uppercase. IRE-like: bold letters. At the 3′ end: Stop codon: Uppercase. PolyA signal: Uppercase, italics and underline. Transcription stop site: brackets. IRE-like: bold letters. CR: Coding region.

Table S2. Ends of sequences coding ribosomal proteins

Annotation or common name	Gene ID	Region	Sequence delineated
Ribosomal protein L3	GL50803_16525	5´UTR/CR	uuccgggucug*aaaag[a]caa*ggccc**gcgAUGucccaccgc**aa**guucucgugcugccgcaagggc**aaccucggcuaccu
Ribosomal protein L4	GL50803_17547	3´CR/UTR	cgccucgaucccagcggaacgguca**ugcccaagUAAaucgcugg**<u>*CGCAAAA*</u>cguu[u]gcacaaugaggagccucgagga
Ribosomal protein L7a	GL50803_17244	5´UTR/CR	uggg*uuca[a]uaaaa*ca**ggccucgacAUGuccaagguuucuggcagcgacauuaagagggcc**cucgccguacccgagaa
Ribosomal protein L7a	GL50803_17244	3´CR/UTR	ggcguucgccgcgagga**ugaggcccagcagaugUAAaugccuua**cauuucuuu<u>*AGUGCAU*</u>guccuu[u]gcugcuugguc
Ribosomal protein L7Ae	GL50803_2825	5´UTR/CR	uggaaaauguaa*uaaauu[a]aaa*cucaauugaaa**caaggAUGccagaugcacgcgcuguucccuug**gccucagaggccc
Ribosomal protein L7Ae	GL50803_2825	3´CR/UTR	acaagcuaaacauauuccuua**cggagcuaggacacUGAuccg**g<u>*AGUAAAU*</u>gguggg[u]gaagcaguguggccugcccauc
Ribosomal protein L7Ae	GL50803_11287	3´UTR	gcacugguauga**cgggagugcguuuacagugcaagcaucacuaguaaaugcucaccg**guacaugcuacacacuacuaguauuugg<u>*AGUGCA*</u>cuaua[u]acgaagg
Ribosomal protein L9	GL50803_17056	5´CR	aauccgcuuucgaugc*ucuuuu[u]uua*gaggucgcgaagcaAUGcc**ccucuguuccuccaugacgaucaagg**ucccgac
Ribosomal protein L10	GL50803_10428	3´CR/UTR	ggaaggggccccucaa**cuugaagcucUGAucggucccaag**caca<u>*AGUAAAU*</u>aauugccaggcauu[u]acuccucugcc
Ribosomal protein L10a	GL50803_1345	3´UTR	uucUAA**gaucuuc<u>*UGAGAAG*</u>guu**cccggggguuu[u]gcau
Ribosomal protein L12	GL50803_14938	5´CR	uuauaguuaaagcauuuua*auuugau[a]uuau*gucagagugguaguuuuccaggcgacaAUGccccc**cagagcugauccuaacgcagagauaguucug**uauuugcgcgucaagggcggg
Ribosomal protein L12	GL50803_14938	3´CR/UTR	uugcugaggagcaagaga**cagagUAAacaaggugcccug**cgugucacggaucggucccuaucugcuugggacuguuuucgccuacggggg<u>*CUUAAAA*</u>uauu[u]cgauggccgaggcggc
Ribosomal protein L13	GL50803_14622	5´UTR/CR	uggaagcucgugcaauugc[a]cuuauaAUGgcaaguuaugcuucuuuuauauugagauuaauucauuuuaauuugaauu
Ribosomal protein L13a	GL50803_11247	5´UTR/CR	uuaaaauuggauuuagcg*caaauc[u]guuu*gcucacggcga**gccAUGucgcgaaucguuaucgacugcaaggggc**acauc

Ribosomal protein L15	GL50803_8001	5´CR	ucauagg*aucauu[u]uuua*cccggccugaauu**cgAUGggugccuacaaguacguucaggagcucuggcg**caagaagcaa
Ribosomal protein L15	GL50803_8001	3´CR/UTR	aaccacacccgcucccuc**cgccgcuacagaUAGccuugcugcugcg***AGUAAAC*aucccgucauu[u]acacuagaggaag
Ribosomal protein L17	GL50803_98056	3´UTR	gaaggcgcuccagUGAgucuguc**gggaguaaaugcgcugcuucu**ucccaacuucu*AAUAAU*ugaaaugucuaaugugggguacauuccgauu[u]cggcccuucccgu
Ribosomal protein L19	GL50803_16431	5´UTR/CR	auuuuucucg*gaacu[u]uuu*cgac**cucccgacAUGgcuaaucugaggcugcagaagcgccuggcggcag**acguccucaa
Ribosomal protein L21	GL50803_15520	5´CR	auuuuaaauuc*auuu[u]caau*gcugcccgaaaagcgaggacgacucgAUG**acgcacagccaggggguacaggugu**aagac
Ribosomal protein L21	GL50803_15520	3´UTR	cgacaucaccccacucaaguaugacggaguuuuc**UAGgcuccgacga*aguaaa*uggccug**cggggcggcu*AGUGGA*ggcuggccu[u]ccaccgguuccucgacgcgcu
Ribosomal protein L23	GL50803_10091	5´UTR/CR	acaacuuuaauuucgc*uuuu[u]acu*cguuuccuaaugacggaucga**ggagAUGgcgucgaagcguggcagguccc**ggagg
Ribosomal protein L23	GL50803_10091	3´CR/UTR	cg**acggcugagugcguccacUAGgc*AGU***_AAAC_gaa[u]uc
Ribosomal protein L23A	GL50803_7870	5´CR	aauuucgccg*ucua[a]uaucu*cgugccggcgagacA**UGuccgagaaggcgca**ggcacugaaggcccaaaaggcgaucuu
Ribosomal protein L24	GL50803_14869	5´UTR/CR	aguguuucucuaccauuuaaauac*uuuaa[a]uaauu*cag**gaacaAUGccucgcauagagcauugcuc**auucugcuccaa
Ribosomal protein L26	GL50803_15046	5´UTR/CR	aaauaauucgauuugaaauggaaauuggccc*auuuuuugaau*cacccggcgaagccug**gaAUGaagcucaacucc**gccgu**caccgcgucccgucgcaagugccg**caaggcuuacuucaca
Ribosomal protein L27a	GL50803_16310	5´UTR/CR	accaaaaaaguauauuaaauuuaauuucauuuuccccacgg*ucu[a]aaaa***ggggccucgagAUGccgacc**agaaucagg
Ribosomal protein L31B	GL50803_16368	5´UTR/CR	ucugaauugucccc*ugcaa[a]aaa*cggguccucc**gaagcgugAUGgguauuguauaugagcauacgauccgccuc**aacaa
Ribosomal protein L31B	GL50803_16368	3´CR/UTR	gcagcgaa**gacggcagcgagUAAacuugugcacgcuc**gcauuuacauaugcuuuuuugc*CGUAAGU*ggg[u]cguccucg
Ribosomal protein L34	GL50803_36069	5´UTR/CR	uuu*uaauuac[u]aaa*gagcugcccgccc**cgagAUGgccgacugucgcgucacuug**ccgcuuccacuccacauacaggacu

Ribosomal protein L34	GL50803_36069	3´CR/UTR	agcuaagacggccaagcagaccgccggcaaggg**gcaugcgaagUAAaucgaucagc**auuuacgucucacuucgggccucuuccgaaccccgccugggacuucauggggaacuucggcuucuggggcu*UUGAAG*cugcuu*[u]*cccccggggccgccugccuugau
Ribosomal protein L35	GL50803_6133	5´CR	ucucgcacaccg*aagag[u]caacuu*gcc**AUGgcauccuguuugccccuaccagauagauguuau**ucaauguauuuuaaa
Ribosomal protein L35	GL50803_6133	3´CR/UTR	cuccaguucccgaagcugaaguaugcu**guaaaggucUGAgcacccugguguuaguaaauac**ucucuugcuucccuuucaccgcguagaacauagcagaugcggagugcguauggcgcacgu*GUGAAA*cucgccagguuu[u]gcaaggguagcgagguggagc
Ribosomal protein L35a	GL50803_5947	3´CR/UTR	gagcgcuuguacgggaaccccaa**cgcgacgguaaccuacgaguaaauagucaggcg**ggcuuguggc*GUGAUG*cugggggucucgcccugguggu u[u]acugcuuugc
Ribosomal protein L36-1	GL50803_16114	5´CR	uuuauuuuccaauccauuuuaaauucauuucaauuccug*auuu*[a]*gaa*ggagcggugugguA**UGccaggcaaaguauucaaccugaagaagggcggcg**caguaguucgcauuguccgca
Ribosomal protein L37	GL50803_14171	5´CR	ccuccaugccggacauagcauuuuaaauuggaauuagucgc*uguu[a]aaaa*cgggaacucgA**UGuccaagggcacagcgucguucgguaagcgccacacgcgc**auucacggcagcugcc
Ribosomal protein L37	GL50803_14171	3´CR/UTR	gcaaacgccaggaagu**accuugccaagUAAauuugcacgaggu**cgguccg*AUGGAUG*ccuacacgc[c]ggugcgucgcg
Ribosomal protein L37a	GL50803_5517	5´CR	ucuggaaauuuaau*aaaau[u]aaauu*gaauucuugcaagcgaaaagcggcucaggcgagcuAUGg**ccaagaggacuaaaaagguuggucucaccggg**aaguacggcacucgcuauggga
Ribosomal protein L37a	GL50803_5517	3´CR/UTR	cacgauccgccgcc**uccgucuggcccgugaagacgcacagUAAaguaaacagaguggga**ccuggcacugcggu*UUGCAA*cugucugcauagcucguu[u]acucgucc
Ribosomal protein L44	GL50803_9810	5´UTR/CR	aaaugcaaauugaauuagcuuugc*augcuacu[u]aa*cagcagcggccgaa**ggacAUGgugacauaucc**ggcagagcgca
Ribosomal protein SA	GL50803_7766	3´UTR	cucuucgacgaguacUAAggcggcucuuguua**agcugaaaguguaaacgcuuugcu**cgguaugcaucccucguuucaccagagagguuucacaacuccaggacgguuccuucugcgauuccgaggauuu*CGUAAA*gucuggcuauca[u]ggcacuacaagugu
Ribosomal protein S2	GL50803_8118	5´CR	uaauuuugauuugacuuuguagccc*aaaac[u]aa*cguccauggcgua**gcAUGcaggcugaaagugcucccaggc**cagaa
Ribosomal protein S4	GL50803_11359	5´CR	ggaugg*uauuc[u]gguuua*gcagcgaAUG**gcccguggaguccgcaagc**accuuaagcgccucaacgcaccgaagcacug

Ribosomal protein S5	GL50803_12981	3´UTR	uauagccguuuccaaccgcUAAacgccc**aggaguaaaccgccccu**cugagagaucggugucaguccucaaa_AAUCAU_auuuggaugaguugcgguu[u]cuccuugcug
Ribosomal protein S6	GL50803_14620	5´UTR/CR	uggugagaaagcauuuuaauuucauuucuag_uuuc[a]uaa_**_aau_ggcccgggggugaAUGccaaagggcucauu**cugcaag
Ribosomal protein S6	GL50803_14620	3´CR/UTR	aagccugcuaa**gaggacgguugagUAAacagacgucuuu**cuacgcgggc_CUGAAUC_uguucgu[u]gcagcguugccuua
Ribosomal protein S8	GL50803_5845	3´CR/UTR	ggaccugagcuucucuucuaccagaa**gaaguuggcaccuaagUAAacaucc**c_AGUGCUU_cgugguc[u]ccccacucuucg
Ribosomal protein S9	GL50803_4547	5´UTR/CR	uauuuu_aaauucaauuu_ggcuuucgccaau[u]cuccccugacga**uuccAUGccccgcauuagccgccauaggaa**cucgu
Ribosomal protein S10B	GL50803_10919	3´CR/UTR	aa**gcaaagUAAaaucugagc**ccauagcuuccacgcacagaugaucccagcggaauguugg_AGUAAAU_caagucc[u]gg
Ribosomal protein S11	GL50803_14827	5´UTR/CR	cuugagagagccgagacucugcgcaaggcugc[u]ua**cgagAUGgcugagaaccagcacgagcg**agcguaucacaagcaa
Ribosomal protein S11	GL50803_14827	3´CR/UTR	gagccuguca**gcgaggccgccaagaagcgcuucucgcccuucUAGgcagcacu**_AG**U**AAAU_aaggg[u]cauucacaaggc
Ribosomal protein S12	GL50803_33862	3´CR	gggacgucgacagcuucc**uuacuaagcuggacgagcagUAA**acgacauacuu_AGUCGUG_cggca[u]cgguagugggauu
Ribosomal protein S13	GL50803_16652	5´UTR/CR	a_uagcaauuu[u]a_gcgggcacgugacaaa**gccAUGggccgcaugcacucgaaggguaaggguauaucccgcucu**gccau
Ribosomal protein S13	GL50803_16652	3´CR/UTR	guccgaccaggccgccgcgcuccuu**gcgacguacgcgUGAguaaacaccgc**cuacggggcucgaacccgcgaccguugg_AUUAAA_aguccaacgcuc[u]accaacugag
Ribosomal protein S14	GL50803_7878	5´CR	uuuugaauugacuuuagaagg_acaaa[u]uu_gaggugggcugaAUG**ucucgcaaaguaaccuacgcgccggaga**aucuuc
Ribosomal protein S15	GL50803_15260	3´CR/UTR	cc**gcugcguUAGucccgcccacc**_**AGU**AAAC_auucc[u]cg
Ribosomal protein S15A	GL50803_15228	5´UTR/CR	cgcacaucgcgugccggcgugcugggaagcaga[u]cca**guaggcAUGguucgcaucaacguucuccgugacgc**acucaa
Ribosomal protein S16	GL50803_4652	5´CR	uuuaauuccacuuccgg_uccca[u]aaaaa_gcggcuccccgAUGgcgaagggcaa**cagaagagacuuacug**gaaaggaac
Ribosomal protein S16	GL50803_4652	3´CR/UTR	uuugguggccguggugcucgugcccgucgccagaaguc**guaccguUAAgcgggcuucguccuaaguaaacauac**ugcauccaauucacacgcugucaucucaccacugacccu

			ggcccuccuggccguuguuguaguaggaggcuuggguguguagccauaugacuggccguacccauuauu*GUGAUC*accauaagcgcugcu[u]ccauaguccugcc
Ribosomal protein S20	GL50803_6022	5´CR	uaaaaug*aauucu[u]uaa*guacgaaaacgcggccaccgaggcuAUGgcagaga**gcgugucgacuggaggcgu**gaaucuu
Ribosomal protein S21	GL50803_7082	5´CR	uaaauugauuuuug*uccuacaa[a]uu*ggacccgaaacgacugag**ccAUGcaaucggaugacaucgg**gaugaagaacagc
Ribosomal protein S21	GL50803_7082	3´CR/UTR	cggucucauggaac**gccuuUAGguugccgcauacaccggccaaguaaacgc**ucugauacuugcuguauuccugcguugauuuacacagccuucggguucagagcgagcaccuggcugcuagguc*GUAAAC*ggccuccaugacagcaucguuu[u]ggggaacc
Ribosomal protein S23	GL50803_14699	3´UTR	cgugcgcguUA**Agcacgggcguguaaacugccacuggcu**gugcauuuu*AGUUUA*cucuucuagcgcuu[u]gagccacacuccauacagaauuucgcugugucugaa
Ribosomal protein S24	GL50803_10367	5´CR	uuuuaaaucugaauuucg*uuguc[a]uuaaa*gcggggcuagcgAUGc**ccgagaucacaguuaaggugaggaagg**uccuca
Ribosomal protein S29a	GL50803_39483	5´UTR/CR	gcgaggaaggcgcgccacggcacgccccg[g]gccc**caggaAUGcgcagugcacuggacaccggcguagcggcug**accgc
Ribosomal protein P1B	GL50803_17337	5´CR	auauccaa*gacaaa[u]aa*ggagcgucacccgagcucguucguAUGcaga**gucaagaaaccgcguguguuuuggc**cgcua
Ribosomal protein P1B	GL50803_17337	3´UTR	agucuuccgacUGAgucugcaugcacaaggacgaggc**uuuacuguaaagg*AGUGAA***cacccgauuu[u]auuguuacgcu

Delineation of the untranslated regions of certain sequences encoding for ribosomal proteins in *G. duodenalis*. At the 5´end: CAATTT element: shading. T/A-rich region: italics. Transcription start site: brackets. Start codon: Uppercase. IRE-like: bold letters. At the 3´ end: Stop codon: Uppercase. PolyA signal: Uppercase, italics and underline. Transcription stop site: brackets. IRE-like: bold letters. CR: Coding region.

Table S3. Ends of sequences coding cytoskeleton proteins

Annotation or common name	Gene ID	Region	Sequence delineated
Actin-related protein	GL50803_8726	3´CR/UTR	ccgaaaccaguugccuu**ugagguuUGAaaggccucg**ugucu*AUGGUAU*au acuauu[u]ccgaugaauaaaucgcu
Actin-related protein	GL50803_16172	3´CR/UTR	ugaaaucuuaa**ugaaagugauaaugUGAcacucuuacaaaagacucuua**gagc ggaucaucguugcccuucauacuugucuc*UGUAAGC*gauccugcccuuu[u] acagcacuu
Actin-related protein	GL50803_11039	3´CR/UTR	gaaaaggcaacaa**agUAAguacauaagaguaaauaacu**aucuaucuaaagaua uauaguguggggugcauguuuuaa*AGUGAA*uccaacagaaccaaaugcggg accuuguauacauauaagccuauaaauaauggaaucauuuu[u]aguuuaua
Hypothetical protein	GL50803_17591	3´UTR	cuggacuggaacacaa**cUGAuuaaaaggauu***AGAAUAA*ccguggaagau[u]cugcuugcuauuuuuggcuuugcu

Delineation of the untranslated regions of certain sequences encoding for ribosomal proteins in *G. duodenalis*. At the 5´end: CAATTT element: shading. T/A-rich region: italics. Transcription start site: brackets. Start codon: Uppercase. IRE-like: bold letters. At the 3´ end: Stop codon: Uppercase. PolyA signal: Uppercase, italics and underline. Transcription stop site: brackets. IRE-like: bold letters. CR: Coding region.

Table S4. Ends of sequences coding metabolic proteins

Annotation or common name	Gene ID	Region	Sequence delineated
Glucosamine-6-phosphate deaminase	GL50803_8245	5´CR	ccucgcc*aaaa[u]aaaa*AUGccguccauccacgucuccaa**augcgcugauccagcca uaaagcuugcccaccguau**c
Pyruvate-flavodoxin oxidoreductase	GL50803_17063	5´CR	cgaagugaaauuacaacauuuugcccc*aauaa[a]uuaa*A**UGucugugguccacgc gccuauagacg**gcugucacgcg
Pyruvate-flavodoxin oxidoreductase	GL50803_17063	3´CR	cuacaagaaguacagcgaaaugcaggcga**ccgccaaggcgg**agUAAgc*AGUGA A*cuuuca[u]uucaugucugggcgg
Thioredoxin reductase	GL50803_9827	5´CR	acgagc*auu[u]uuaa*AUGuccacucagcgccacgucaggaucggaaucaucggcg**g aggcccugcaggccuc**acugc
Thioredoxin reductase	GL50803_9827	3´CR/UTR	aaggcggcacucgacgccgagcgcug**gcuugccaugcaggagUA*AGU***_AAA_ugg cagcgggccug[u]ggcuccaguucagccgaaaggauagcugggggaggggaugu

Delineation of the untranslated regions of certain sequences encoding for ribosomal proteins in *G. duodenalis*. At the 5´end: CAATTT element: shading. T/A-rich region: italics. Transcription start site: brackets. Start codon: Uppercase. IRE-like: bold letters. At the 3´ end: Stop codon: Uppercase. PolyA signal: Uppercase, italics and underline. Transcription stop site: brackets. IRE-like: bold letters. CR: Coding region.

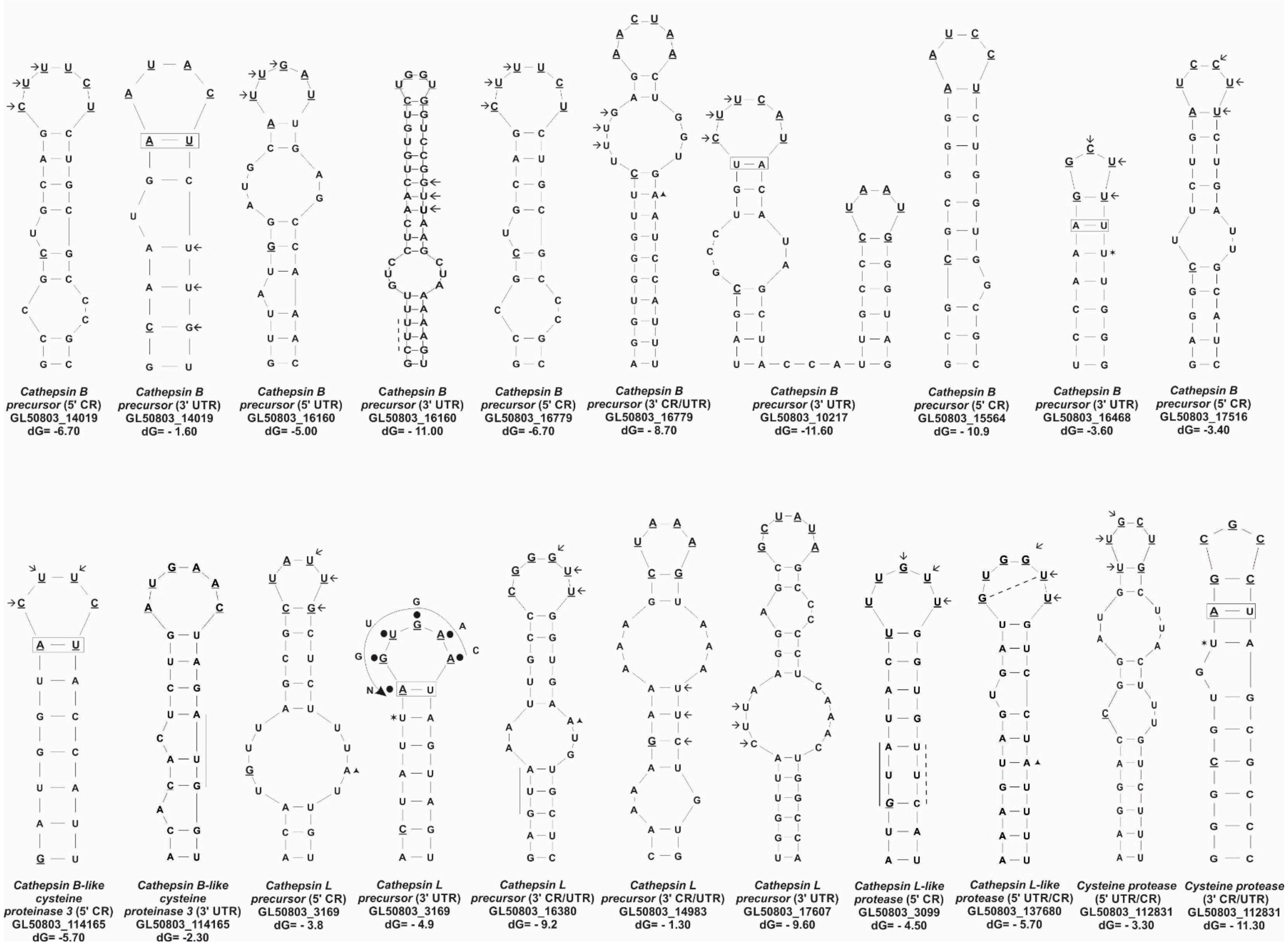
Cathepsin B precursor (5' CR) GL50803_14019 dG= -6.70
Cathepsin B precursor (3' UTR) GL50803_14019 dG= - 1.60
Cathepsin B precursor (5' UTR) GL50803_16160 dG= -5.00
Cathepsin B precursor (3' UTR) GL50803_16160 dG= - 11.00
Cathepsin B precursor (5' CR) GL50803_16779 dG= -6.70
Cathepsin B precursor (3' CR/UTR) GL50803_16779 dG= - 8.70
Cathepsin B precursor (3' UTR) GL50803_10217 dG= -11.60
Cathepsin B precursor (5' CR) GL50803_15564 dG= - 10.9
Cathepsin B precursor (3' UTR) GL50803_16468 dG= -3.60
Cathepsin B precursor (5' CR) GL50803_17516 dG= -3.40
Cathepsin B-like cysteine proteinase 3 (5' CR) GL50803_114165 dG= -5.70
Cathepsin B-like cysteine proteinase 3 (3' UTR) GL50803_114165 dG= -2.30
Cathepsin L precursor (5' CR) GL50803_3169 dG= - 3.8
Cathepsin L precursor (3' UTR) GL50803_3169 dG= - 4.9
Cathepsin L precursor (3' CR/UTR) GL50803_16380 dG= - 9.2
Cathepsin L precursor (3' CR/UTR) GL50803_14983 dG= - 1.30
Cathepsin L precursor (3' UTR) GL50803_17607 dG= - 9.60
Cathepsin L-like protease (5' CR) GL50803_3099 dG= - 4.50
Cathepsin L-like protease (5' UTR/CR) GL50803_137680 dG= - 5.70
Cysteine protease (5' UTR/CR) GL50803_112831 dG= - 3.30
Cysteine protease (3' CR/UTR) GL50803_112831 dG= - 11.30

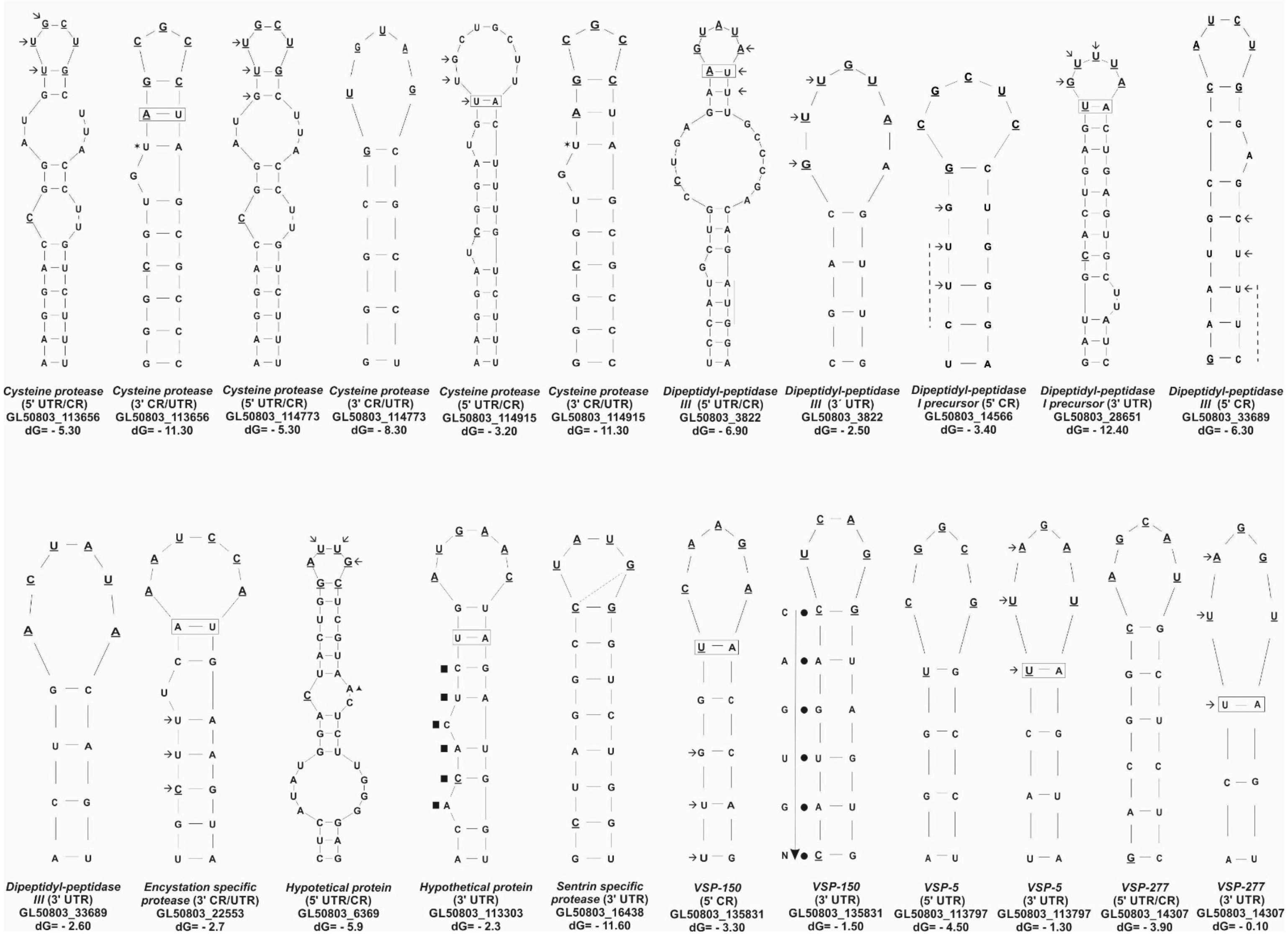
Cysteine protease (5' UTR/CR) GL50803_113656 dG= - 5.30
Cysteine protease (3' CR/UTR) GL50803_113656 dG= - 11.30
Cysteine protease (5' UTR/CR) GL50803_114773 dG= - 5.30
Cysteine protease (3' CR/UTR) GL50803_114773 dG= - 8.30
Cysteine protease (5' UTR/CR) GL50803_114915 dG= - 3.20
Cysteine protease (3' CR/UTR) GL50803_114915 dG= - 11.30
Dipeptidyl-peptidase III (5' UTR/CR) GL50803_3822 dG= - 6.90
Dipeptidyl-peptidase III (3´ UTR) GL50803_3822 dG= - 2.50
Dipeptidyl-peptidase I precursor (5' CR) GL50803_14566 dG= - 3.40
Dipeptidyl-peptidase I precursor (3' UTR) GL50803_28651 dG= - 12.40
Dipeptidyl-peptidase III (5' CR) GL50803_33689 dG= - 6.30
Dipeptidyl-peptidase III (3' UTR) GL50803_33689 dG= - 2.60
Encystation specific protease (3' CR/UTR) GL50803_22553 dG= - 2.7
Hypotetical protein (5' UTR/CR) GL50803_6369 dG= - 5.9
Hypothetical protein (3' UTR) GL50803_113303 dG= - 2.3
Sentrin specific protease (3' UTR) GL50803_16438 dG= - 11.60
VSP-150 (5' CR) GL50803_135831 dG= - 3.30
VSP-150 (3' UTR) GL50803_135831 dG= - 1.50
VSP-5 (5' UTR) GL50803_113797 dG= - 4.50
VSP-5 (3' UTR) GL50803_113797 dG= - 1.30
VSP-277 (5' UTR/CR) GL50803_14307 dG= - 3.90
VSP-277 (3' UTR) GL50803_14307 dG= - 0.10

Figure S1. Prediction of stem-loop structures of *G. duodenalis* mRNAs coding for virulence proteins. The six nucleotides that form the apical loop of the canonical IREs are underlined. The dotted line in the loop indicates the residues at position 1-5 that form a pseudotriloop (G-C/C-G or G-U). Nucleotides U-A/A-U in rectangle indicates conserved nucleotides in the upper stem. Filled black circles indicate similar nucleotides to the CAGUGN consensus human motif and a large, curved arrow indicates the 5′ to 3′ or 3′ to 5′ orientations of the sequence. The GUU/UUG protozoa-specific motif is indicated in small arrows. Filled black squares show residues similar to that reported for the *tvcp4*-IRE loop. Asterisk: residue U conserved on canonical IREs. Small triangles show the residue A conserved in protozoa-specific motif. The residue C or G underlined indicates the unpaired residue at position 8. dG, Gibbs free energy or free enthalpy; in this case, a reduction in dG (negative dG) is a necessary condition for the spontaneity of the structure formation.

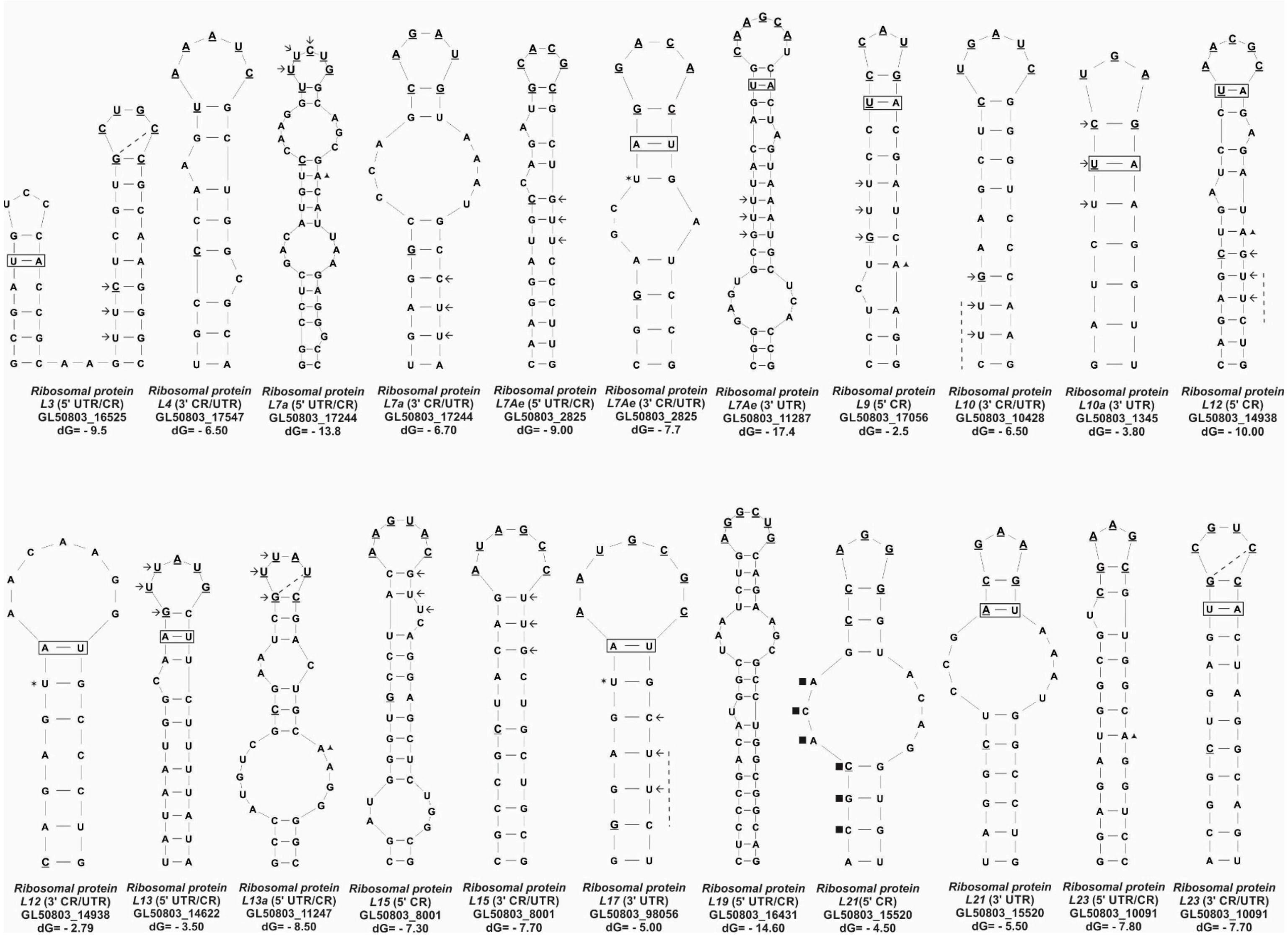

Ribosomal protein L3 (5' UTR/CR) GL50803_16525 dG= - 9.5
Ribosomal protein L4 (3' CR/UTR) GL50803_17547 dG= - 6.50
Ribosomal protein L7a (5' UTR/CR) GL50803_17244 dG= - 13.8
Ribosomal protein L7a (3' CR/UTR) GL50803_17244 dG= - 6.70
Ribosomal protein L7Ae (5' UTR/CR) GL50803_2825 dG= - 9.00
Ribosomal protein L7Ae (3' CR/UTR) GL50803_2825 dG= - 7.7
Ribosomal protein L7Ae (3' UTR) GL50803_11287 dG= - 17.4
Ribosomal protein L9 (5' CR) GL50803_17056 dG= - 2.5
Ribosomal protein L10 (3' CR/UTR) GL50803_10428 dG= - 6.50
Ribosomal protein L10a (3' UTR) GL50803_1345 dG= - 3.80
Ribosomal protein L12 (5' CR) GL50803_14938 dG= - 10.00
Ribosomal protein L12 (3' CR/UTR) GL50803_14938 dG= - 2.79
Ribosomal protein L13 (5' UTR/CR) GL50803_14622 dG= - 3.50
Ribosomal protein L13a (5' UTR/CR) GL50803_11247 dG= - 8.50
Ribosomal protein L15 (5' CR) GL50803_8001 dG= - 7.30
Ribosomal protein L15 (3' CR/UTR) GL50803_8001 dG= - 7.70
Ribosomal protein L17 (3' UTR) GL50803_98056 dG= - 5.00
Ribosomal protein L19 (5' UTR/CR) GL50803_16431 dG= - 14.60
Ribosomal protein L21(5' CR) GL50803_15520 dG= - 4.50
Ribosomal protein L21 (3' UTR) GL50803_15520 dG= - 5.50
Ribosomal protein L23 (5' UTR/CR) GL50803_10091 dG= - 7.80
Ribosomal protein L23 (3' CR/UTR) GL50803_10091 dG= - 7.70

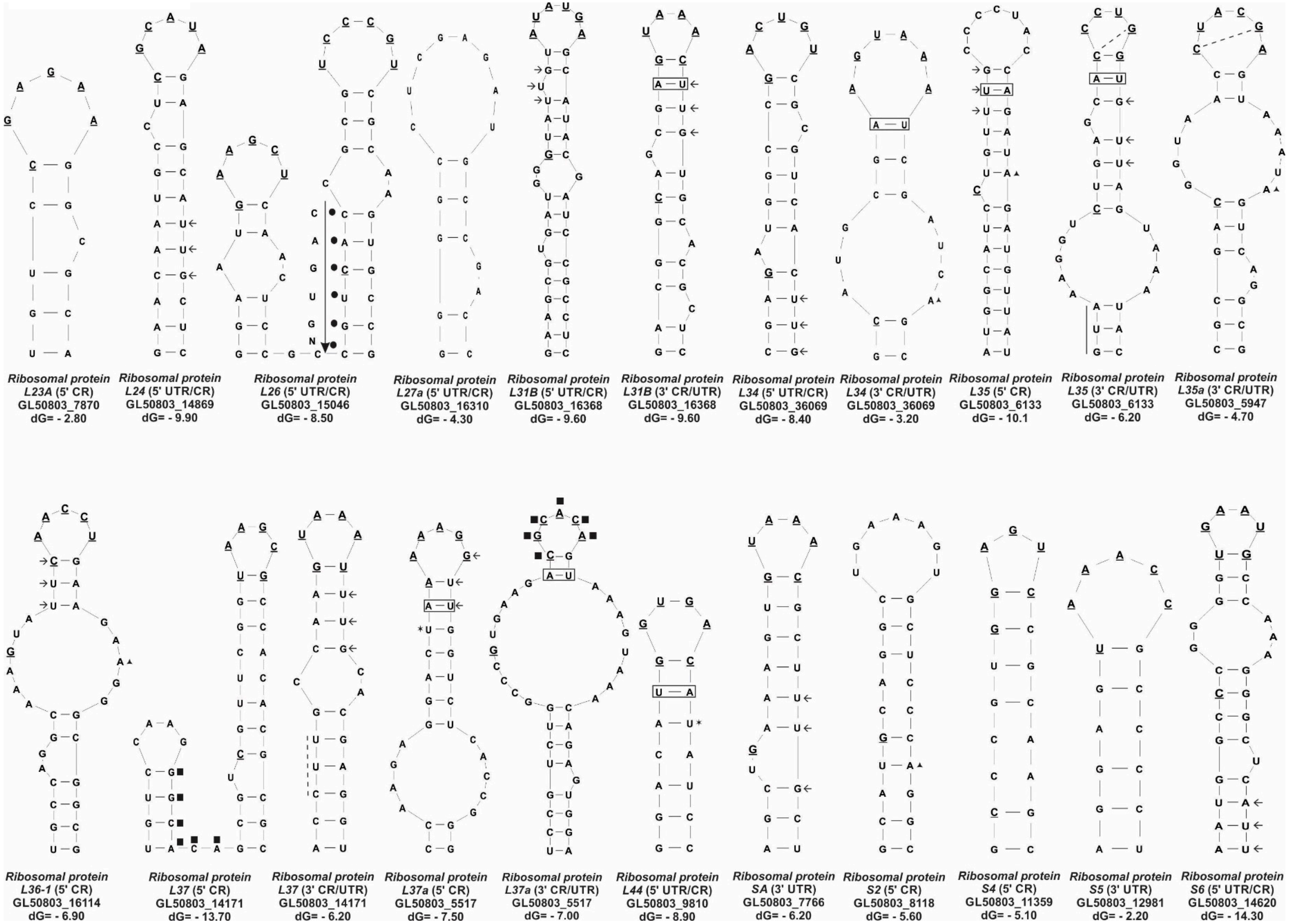

Ribosomal protein *L23A* (5' CR) GL50803_7870 dG= - 2.80
Ribosomal protein *L24* (5' UTR/CR) GL50803_14869 dG= - 9.90
Ribosomal protein *L26* (5' UTR/CR) GL50803_15046 dG= - 8.50
Ribosomal protein *L27a* (5' UTR/CR) GL50803_16310 dG= - 4.30
Ribosomal protein *L31B* (5' UTR/CR) GL50803_16368 dG= - 9.60
Ribosomal protein *L31B* (3' CR/UTR) GL50803_16368 dG= - 9.60
Ribosomal protein *L34* (5' UTR/CR) GL50803_36069 dG= - 8.40
Ribosomal protein *L34* (3' CR/UTR) GL50803_36069 dG= - 3.20
Ribosomal protein *L35* (5' CR) GL50803_6133 dG= - 10.1
Ribosomal protein *L35* (3' CR/UTR) GL50803_6133 dG= - 6.20
Ribosomal protein *L35a* (3' CR/UTR) GL50803_5947 dG= - 4.70
Ribosomal protein *L36-1* (5' CR) GL50803_16114 dG= - 6.90
Ribosomal protein *L37* (5' CR) GL50803_14171 dG= - 13.70
Ribosomal protein *L37* (3' CR/UTR) GL50803_14171 dG= - 6.20
Ribosomal protein *L37a* (5' CR) GL50803_5517 dG= - 7.50
Ribosomal protein *L37a* (3' CR/UTR) GL50803_5517 dG= - 7.00
Ribosomal protein *L44* (5' UTR/CR) GL50803_9810 dG= - 8.90
Ribosomal protein *SA* (3' UTR) GL50803_7766 dG= - 6.20
Ribosomal protein *S2* (5' CR) GL50803_8118 dG= - 5.60
Ribosomal protein *S4* (5' CR) GL50803_11359 dG= - 5.10
Ribosomal protein *S5* (3' UTR) GL50803_12981 dG= - 2.20
Ribosomal protein *S6* (5' UTR/CR) GL50803_14620 dG= - 14.30

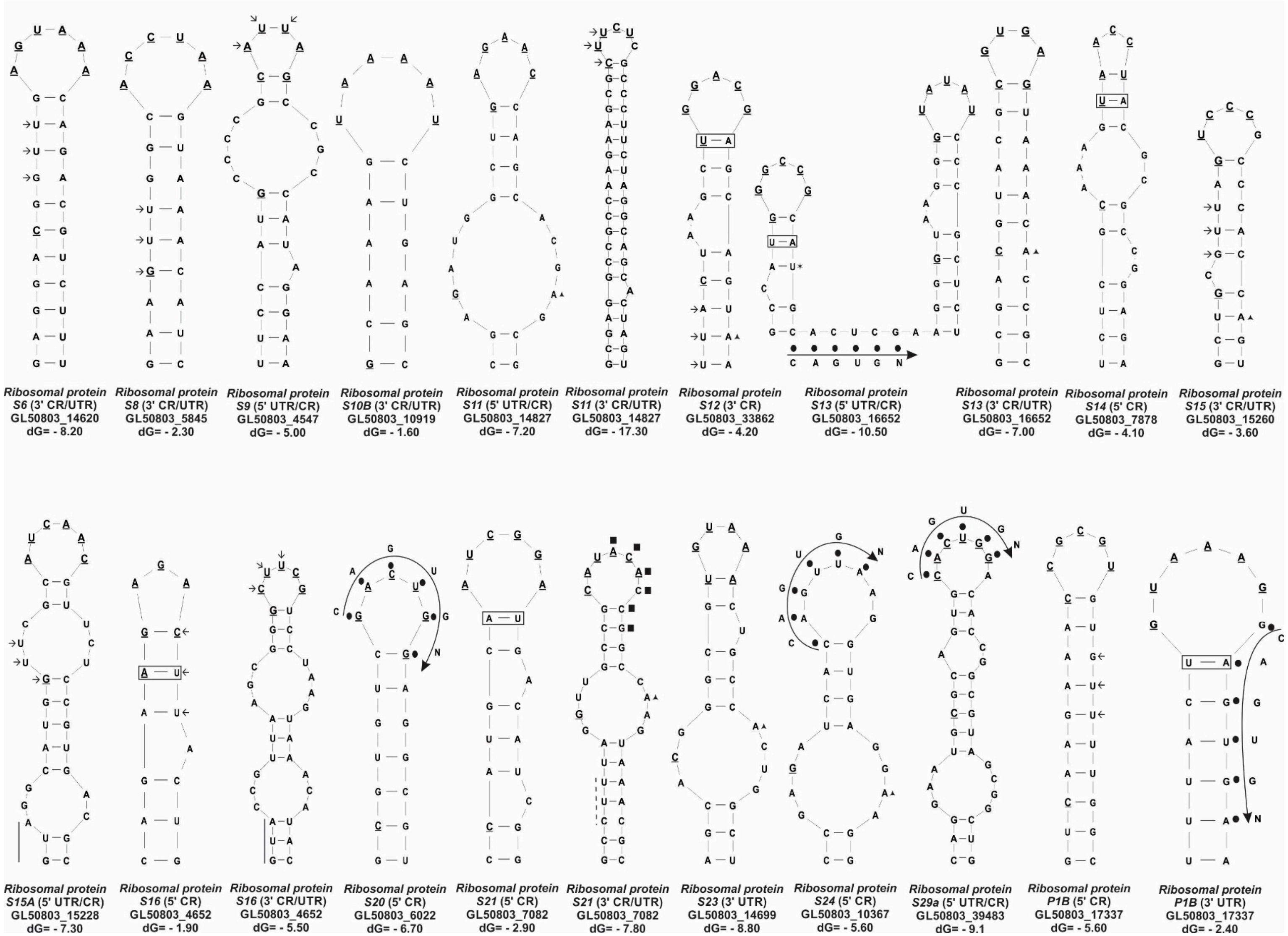
Ribosomal protein S6 (3' CR/UTR) GL50803_14620 dG= - 8.20
Ribosomal protein S8 (3' CR/UTR) GL50803_5845 dG= - 2.30
Ribosomal protein S9 (5' UTR/CR) GL50803_4547 dG= - 5.00
Ribosomal protein S10B (3' CR/UTR) GL50803_10919 dG= - 1.60
Ribosomal protein S11 (5' UTR/CR) GL50803_14827 dG= - 7.20
Ribosomal protein S11 (3' CR/UTR) GL50803_14827 dG= - 17.30
Ribosomal protein S12 (3' CR) GL50803_33862 dG= - 4.20
Ribosomal protein S13 (5' UTR/CR) GL50803_16652 dG= - 10.50
Ribosomal protein S13 (3' CR/UTR) GL50803_16652 dG= - 7.00
Ribosomal protein S14 (5' CR) GL50803_7878 dG= - 4.10
Ribosomal protein S15 (3' CR/UTR) GL50803_15260 dG= - 3.60
Ribosomal protein S15A (5' UTR/CR) GL50803_15228 dG= - 7.30
Ribosomal protein S16 (5' CR) GL50803_4652 dG= - 1.90
Ribosomal protein S16 (3' CR/UTR) GL50803_4652 dG= - 5.50
Ribosomal protein S20 (5' CR) GL50803_6022 dG= - 6.70
Ribosomal protein S21 (5' CR) GL50803_7082 dG= - 2.90
Ribosomal protein S21 (3' CR/UTR) GL50803_7082 dG= - 7.80
Ribosomal protein S23 (3' UTR) GL50803_14699 dG= - 8.80
Ribosomal protein S24 (5' CR) GL50803_10367 dG= - 5.60
Ribosomal protein S29a (5' UTR/CR) GL50803_39483 dG= - 9.1
Ribosomal protein P1B (5' CR) GL50803_17337 dG= - 5.60
Ribosomal protein P1B (3' UTR) GL50803_17337 dG= - 2.40

Figure S2. Prediction of stem-loop structures of *G. duodenalis* mRNAs coding for ribosomal proteins. The six nucleotides that form the apical loop of the canonical IREs are underlined. The dotted line in the loop indicates the residues at position 1-5 that form a pseudotriloop (G-C/C-G or G-U). Nucleotides U-A/A-U in rectangle indicates conserved nucleotides in the upper stem. Filled black circles indicate similar nucleotides to the CAGUGN consensus human motif and a large, curved arrow indicates the 5′ to 3′ or 3′ to 5′ orientations of the sequence. The GUU/UUG protozoa-specific motif is indicated in small arrows. Filled black squares show residues similar to that reported for the *tvcp4*-IRE loop. Asterisk: residue U conserved on canonical IREs. Small triangles show the residue A conserved in protozoa-specific motif. The residue C or G underlined indicates the unpaired residue at position 8. dG, Gibbs free energy or free enthalpy; in this case, a reduction in dG (negative dG) is a necessary condition for the spontaneity of the structure formation.

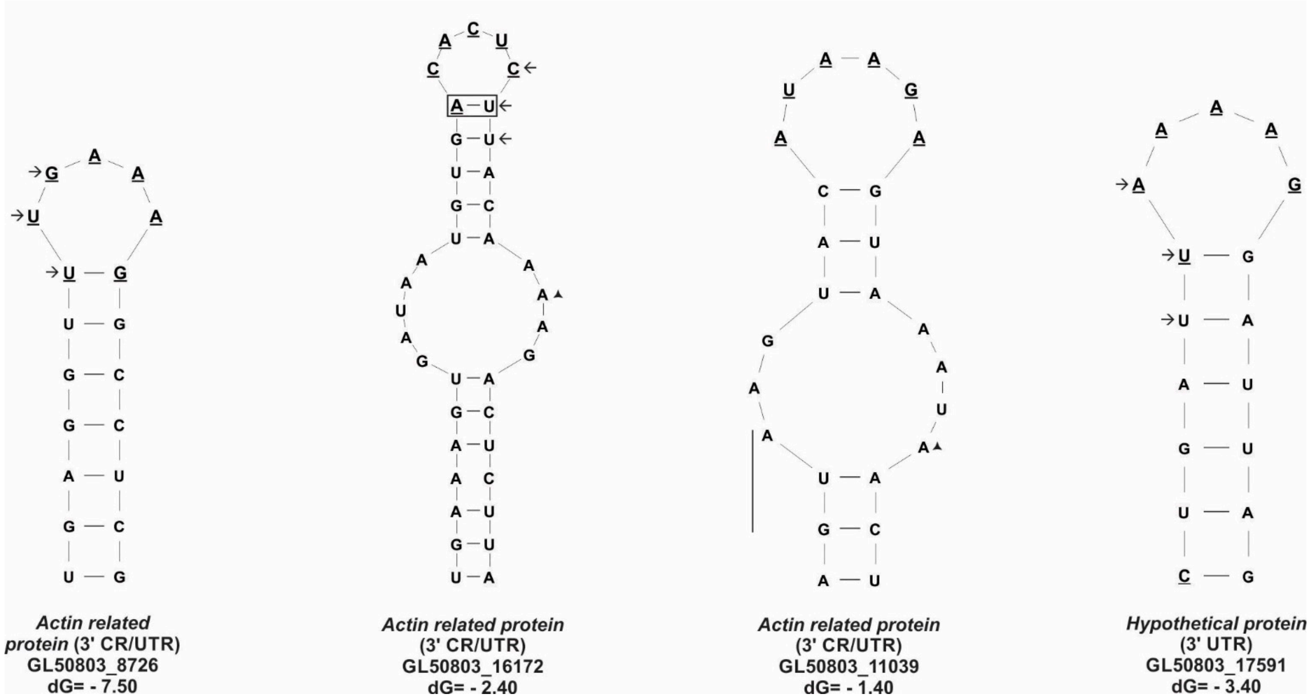

Figure S3. Prediction of stem-loop structures of *G. duodenalis* mRNAs coding for cytoskeleton proteins. The six nucleotides that form the apical loop of the canonical IREs are underlined. The dotted line in the loop indicates the residues at position 1-5 that form a pseudotriloop (G-C/C-G or G-U). Nucleotides U-A/A-U in rectangle indicates conserved nucleotides in the upper stem. The GUU/UUG protozoa-specific motif is indicated in small arrows. Small triangles show the residue A conserved in protozoa-specific motif. dG, Gibbs free energy or free enthalpy; in this case, a reduction in dG (negative dG) is a necessary condition for the spontaneity of the structure formation.

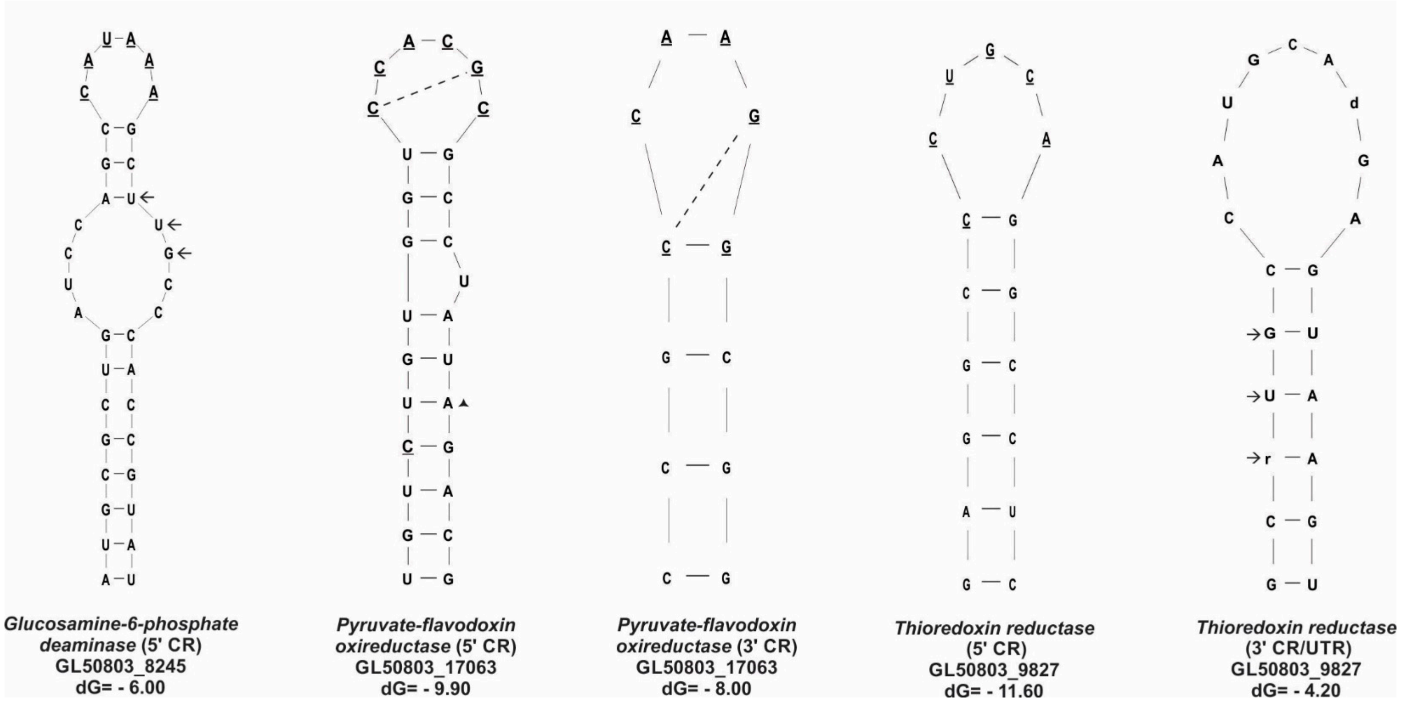

Figure S4. Prediction of stem-loop structures of *G. duodenalis* mRNAs coding for metabolic proteins. The six nucleotides that form the apical loop of the canonical IREs are underlined. The dotted line in the loop indicates the residues at position 1-5 that form a pseudotriloop (G-C/C-G or G-U). Nucleotides U-A/A-U in rectangle indicate conserved nucleotides in the upper stem. The GUU/UUG protozoa-specific motif is indicated in small arrows. Small triangles show the residue A conserved in protozoa-specific motif. dG, Gibbs free energy or free enthalpy; in this case, a reduction in dG (negative dG) is a necessary condition for the spontaneity of the structure formation.

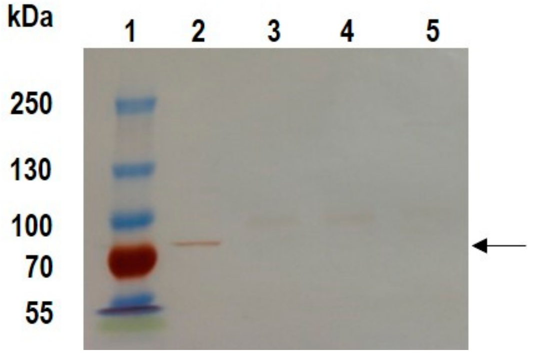

Figure S5. Immunodetection of a cytoplasmic protein in *G. duodenalis* by an anti-human IRP-1 antibody. Western blot assays using cytoplasmic extracts of parasites grown in different iron conditions and interacting with the anti-human IRP-1 antibody. Lane 1. Molecular size markers. Lane 2. Cytoplasmic extracts under iron-restricted condition. Lane 3. Cytoplasmic extracts in normal iron concentrations. Lane 4. Cytoplasmic extracts in high iron concentrations (200 μM). Lane 5. Cytoplasmic extracts in high iron concentrations (300 μM).

Table S5. IRE-like structures predicted by SIRES web server of some mRNA from *G. duodenalis*

Annotation or common name	Gene ID	IRE Sequence
Pyruvate-flavodoxin oxidoreductase	GL50803_114609	TGCCTGCCACGCGCAGAGCTACGTGAGCAAG
Cathepsin L precursor	GL50803_3169	GAAAGGTTCGCAACCAGGGGGTCTGCGGGTGCT
Cathepsin L-like protease	GL50803_3099	GTAATTCCCACTGTAGAATTGGAGGAAAACT
Hypothetical protein	GL50803_113303	CCTTCCCCCTTGCGGGAGGGTGACGACCAGA
Ribosomal protein L7a	GL50803_17244	TCTTATGCGCGAGGAGCCGTCCGCTGACCGC
Ribosomal protein L12	GL50803_14938	GGTCTTCCGCCCAAAGTTGTTGGTGAGAAGA
Actin-related protein	GL50803_8726	AGCAGAGCCAGGTGGGAGCATCTAGCATGCCG

Figure S6. Attached material showing the sequences of some mRNAs presented in our study and analyzed by SIRES web server.

>Pyruvate-flavodoxin oxidoreductase (GL50803_114609)1-4002

CCAATGGGCTAGCGAAAACCCTGGTGCCCCCTATGGTCTGGAGCAAGGCCCAAACGGGTA
ACTTAATTTAAAGCTTAAACGCCTGTGGTCAAGACATTTCAGAGGCCACAAAATTAAAAT
ATGACGACCGCCCTGTCCTCTCAGGAGCAGCTGTGCATCGACGGCGCGACTGCGACGTCC
ATGATGGTGTACAGGCTGATCGACTTCACGGCCATCTTCCCCATCACCCCGTCCTCTGTC
ATGGCCGAGATGATCGAGCAGTACGCCGCGCAAGGCAAGAAGAACATCTTCGGAAACGTC
GTCACTGTCCGCCAGGCCTGGTCGGAGCTTGGAGCCATTGGCACGTGTCACGGAGCAGCG
ACCGCAGGCGCCCTCACGGCGACCTTCAGCTCGTCGCAGGGGGTCCTCCTCATGATCTCT
AACCTCTACCACATGGCTGGCGAGCGCATCCCCTTCGTCCTGCACGTCGCCACGCGCACC
GTCGGGATGGCAGCCACCTCTCTCGCTACAGATCATACGGACGTCTACGCAATCGAAGGC
ACGAACATGTGCGCCCTGTCCAGCTGCAACGTCCAGGAGTGCTACGACATGTCTCTGGTT
GCCCACACGACTGCTACGAATGTCCAGGCCGCCTTCTGCCATTTCTTCGAGGGATATCGT
GTCTCCCACCAGTTCGAGACGATCCAGGTCTTCAAGGACGACGCAGACATCAAGGACTTC
CTCAGCGAGGACCAGCTTGCCAAGTGGAGGAGCGAGAACGCCATGGACACTGATCGCCCG
ATCGCCTCGAACATCGGTCTCGGAGGCGAGATCCACATGCAGATAGTTGAGTCCCAGGGT
GCTGTCTTCGACTCTATCCCGTACCGTATCGAGGAGGCCTTCGACCTGCTCCACCGGAAG
ACTGGCCGCCGTTACCACTGCTTCGAGTACTTCGGCCACCCGACCCCCGATGTTGTTGTC
GTCGTCATGGGCGCAACCAGCCTCACTTTTGAAAACGTCGTCGACGCGCTTGTTAACCGC
GAGAACCGCCGCGTTGGCTTTGTCAGAGTCCGCCTCTTCAGGCCGTTCAGCATCGAGCTC
TTCAGCAACGCTATTCCCAAGTCTGCGAAAGTTGTTGTGGCCCTCGATCGCGCACCAGAG
CTCCTGAACGCCGGCGGTACGCTCTACCGCGAGACTCTTGCCGCTCTCATGAAGACTGGC
CGCCTTGGCGCGGGCAAGAACTCTGTCCGCCTCGTTGCTGGCGGCCGCTACTCGTACTAC
GGATGGGAGCTCGGGCCCGCAGACGTGGTCACTCTCTATGAGACGTTCGACGTCAGCACG
CGCCACGTCGAGAAGATGCCGACGGACTTCGTGCTCAGCATCAAGGACGATATCCGCAAG
CGCTCTCTCCGCCCGGCGCTCCCGGAGCACGTCCCGAAGAGCCTCCTTCCAGAGGGCACC
ACGGAGTGCATCCTCTGGGGTCTCGGCTCAGACGGCACCATTGGTGCCTGCCGCAACGCC
ATGAAGATCCTCTCCGACCGCGTGGGGTGCGAATGCCAAGCCAACTTCGAGTTCGACGGC
AAGAAGTCCGGCGGCACCACCGTCTCACACCTTCGCTTCGGGCCAAAGAAGATCAGGGCC
CAGTACAACATCGAGGAGGCCGGGTATGTTGCCTGCCACGCGCAGAGCTACGTGAGCAAG

TTCAACGTCCTCCACGGCATCAAGGAGGATGGGTTCTTTGTCCTGAACACGGAGCATGAT
ACGGTCGAGACTCTCGAGAAGTATCTGCCTGCCGAGATGAAGAGGGAGATTGCCCGCAAG
AACATCCGAGTCTATGCCGTCAACGCAAATAAGGTCGCGCAGTCGGTCGGCCTTGGCGGA
CGCATAAACACGATCATGATCCTCTTCTTCCTCAAGCTCGGGCTCAGCAAGCTTCTTGAC
TTCGATGTCGCCTGCGAGGACATGAAGGCTGCCGCGAGGATTACCTACGCTAAGCAGAAG
GCAGAGGTCATCGAGGCGAACGTGAAGGCCATTGACGTTGCTCGCTCCATTATAGAGCAG
TGCCACATAGAGTACGATAAGGCGCGCTGGGTCAACGCTGACCCGAGCGAGTCGACTGCA
AACCAAACCTACGGCCCAGAGCCTGACAAGTATGTCAAGGACATCATCTTGCCTGCCGTC
ACGAGGAAGTTCGCCAACATCGGCACCAAGGATGTGATGAAGTACAACTCTGGGAAGATT
CCAGGCGGCTACTCCAAGTATGAGAAGCTCGGCTCTGCCGCCAAGGTTCCCATGTGGAAG
AAGGAGAACTGCATCCAGTGCGGTTTCTGCTCTGTCCAGTGCCCGCACGCCGCCGTCCGC
TGCTTCGTCATGAATGAGGACGCAAAGCCTGACAACGTCCCCGAGGACTTCGAGATGCTC
GACATGAAGGGCAAGCTCGCTGCCGTTAACAGCGATTCCTCGAAGATCAAGTTCCGCGTC
CAGGTTTCGCCGCTCGACTGCAGGGGATGCGGAGTCTGTGTAGAGGCCTGTCCCAAGGAT
GCCCTTGCCATGACCCCCATCGACACTGTCCTCGACAAGCAGCAGAAGCTCTTCGACTGG
GCCTACGATTCTCTTCAGTTCACCCAGGGCACCACAAACATCGACACGGAGAGCTGCAAC
CTCCGCGACCTGCAGCTCCGCTTCCCGTACATGGAGTTCCCTGGCTCCTGCCCCGGCTGC
GGTGAGAGTAACACGATGAAGATGCTGGCGACCCTCTATGGAGATTCGATGGTCGCCTCC
ATCGGCGTCGGGTGCTCGCTTGTCTGGATGCATTTCGGCTACATGCGTCCGTTCAACCTT
GACAGCGACTCCCGCGGTATTGCCGCCTGCTCGTCGCTCTTTGAGGACAACTCTGTTTTC
GGCTGGGGTGTCGCTCTTGATCGCGATGCCAAGCGTGCAAACCTTCGCGAGTACCTTCAA
GCCAACGTCGAGAAGATCACTGACGAGTCCCTGAAGACTACCGTCCAGGGCTGGCTCGAC
GCGTTCGACGACCGCAAGGCCTCCATCCGCACGTCTCGTGAGCTCCGCAGCACGGTCGGC
ATCGAAAGGCTCGACAAGGGCGACTACAAGAACAACCCTGAGTATATCATTCCGACTGTG
GACAATCTTGCTTCCAAGTTCCAGCAGTCCGGCGTCGATCTGGATGTTGCGAAGCACATC
CTCCTCAACAACAACTACCTTATCAAGGCCTCCCATTGGCTTGTGGGCGGTGACGGCTGG
GCCTACGACATTGACTTTGCTGGCTTCGACCACATCGTCGCCTCTCGCGATCGCGTGCGT
GTGCTCGTTCTCGTCAACCACTGCTTCGGTAACACGGGTGGCCAGAAGTCGAAGGCCACG
TCGATCGGCAGCGTCATGAAGAACGCGTACTCCGGCGTCAACGTCAAGGACAAGAAGATG
GGCGTCATGCTCATGAGCACATACCGCGACGTCTACGTGGCACAGATCTCCCTCGGCTAC
AACCGTAACCAGGCCCTCCAGGTCTTCAGGGAGGCAGAGCACTTTGACGGCCCCGCAGTT
GTTCTCGCCTACTGCCCGTGCATCTCGCACATGGTCCAGGGCGGTCTCACTCACCAGGAG

AGGCAGCAGAAGCTCGCCGTCGAGACGGGTGTCTGGCCTGTCTTCCGCTACAACCCGTCC
AAGCCCATCGGTGACCGCATGAAGCTGGAGTACAAGGGGCCAATCAAGCCCGTCAAGGAC
TTCATGAACACGGAGCAGAGGTTCGGCGCCCTTGCAAAGAGGGACCCTGCCAGGTTCGAG
GAGATGGCTGCGGCTCTCCAGTCCGACGTTGAGGAGCACTGGAAGGTTCTTCAGGGGCTC
AAGGCCATCGACGTGCCCACGGACCCGATTGTTTTCGGGTAAAAAAGTGAATCATCTCGC
GATGCTTTTTCGGGGGGTCTTGTACGTCTCTTACGGTATCCACCTTGCTCTCCACGCTCT
TCTGTTGCTGCTCCTCGTGGCCCAGCTCCTTCTTGTCTTCCA

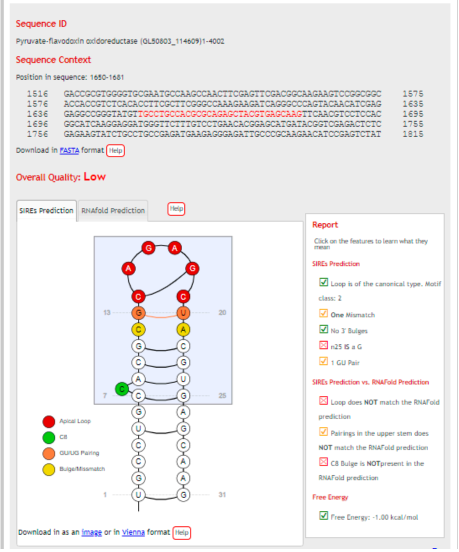

>Cathepsin L precursor (GL50803_3169)1-1935
AAATACTTCAAAAAAGTGTTATGCCAATATGGCATAAAATATTAAAACTGTGGAAATTTC
ATTCTGAATTTCTCATTGAGGTAACTTAATAGTTACAAACTGCAATCTAAGACATCTCAC
ATGTTAGCGCTATTGCTCTTTATTGTCTGCTGTGTTTCCCATGATCCGAGTAACATGGCC
TTTTATATTGACCCCGATAAAATCACATTTGAGACAATTCATGAAAACACAAAGACCCTG
TTTTCTCTTTTCAACAGTCGTTTTACTTTAGACTATCCCTCAGATGAGGCTCACAACAAA
GCCTTGCTGTCTTTCGCGAAAAATCTAGCACACCACAATGCGACCCATGCGAGGGACACG
GACCACCTGGACGAATTGTTGAGGTATACAGATGGATATGCAACATATGGGCTGACATCA
GCCTTTGCCAGAGACCCAACTGAGCCCATGAAGGGATTTGTCCAGGACACCGGTGTCCCC

GAGTACGACTTGAGCAGCTTTAAGGACAGAATACGCTCAAGATTGAATCTGTTCAAGGAA
GCAGCCGCTTATCAGCTCAAGGTCATCTACTCTGCACTAGTGTCGGCTGCACTTCCGGCC
CCTCGAAGTGGATGCACTACGATGTACATACGGCAAAACCCCCTTTCTCCTTTATCATCG
ATTCCGTTGTCTGCGGATTTGCGCGAATTCGAGGTTATAGGAAAGGTTCGCAACCAGGGG
GTCTGCGGGTGCTGCTGGGCAATGGCCTCAGCTGTCATGCTAGAGGGTATGACCCGGTCC
ACACAAAGTTACTTTAAAGACAAAACGGAAGTTGACAACACGTTCAAAGATGTAGACCTT
ATTGTTTCCGAGCAGTACATAATGAACAACTCTCGCAGCTCTATCAACAACTTCTGTGAA
GGTGGAAACTACAGGATCACATCGATGGATTATGCCAATGAAAACATAGTTAAGACAGTG
GAGTCTGAAAAGAATTTTCCACTAGAATCGACTCACTTGGAGAAGAATCCATCAAGAATT
GACGTTATAGCTAATTTCAAAGTGTCCAAGGCCTTCTTGCCTTACAACACAGAGACAGTG
ACAGGGACCAACTGCAAAAGGTCTATCATTAAGCTGCTGGACAAGATGCAGACCTCGTCG
CCAATGACAAACGGGGCCGTCAATCAAATCAAGTCTTACCTTGCACGGGGGATCCCTGTT
GCCGCGGCCATGTTTACGGGGGCAGACGGGCCTCTGTCCTCACGAGACTTCAGCAACTAC
AAGGGCGGCATTTTCAATAAGCCGTGCAGCAAAACAGGGCTTGACCACCAAGTCATGTTT
GCGGGATATGGATATTACCAGGGAGTTGAGGTCTGGGTCATGCGCAACTCCTGGGGAGAA
CAGTGGGGGTCATACGGCCACTTCTACACACCGATAGGCAACAACGTTCTTTGCTCTGAG
ACGTACGCATATACAGACATACCACAGTACTTTCCATTAGACAAAGCTGATAAGGACAAG
CCATACACAGAAAGGCAAAAAACGAAAGCTTCTACCTACTGGGCGAGAAAATTAAAACGT
GGAGATGGTGTAGCCCTCGACTCAGACCCCTCTACGATACCACCAGCCAGTCCAGGCAAT
GATAATGACATATATGTTCCGAGAGGGAAGATCATTTCGTCTTCAAGCAGTGGAACTAGT
AGTAGCAGCAGTAGCACTACTATCACGGGCAAGTTTAAACTTTCAATCCCGAGTATTATC
ATGATTGTGACGGGCATCCTGGGGAGCGCAGGCGTTGCAGGCATTATCGCAAAGTTCACC
TGCTGCAGGAAGTAGGTGCGGATGACTTCTAACTATTAGTGAATAGTAGTGAATAGTAAT
AGACTAGTGGACAACTTAGGATTGATTTTTTTTCTTTGCAATCTTCTCTCTACAAACAAT
TTTAAATTGGAATTT

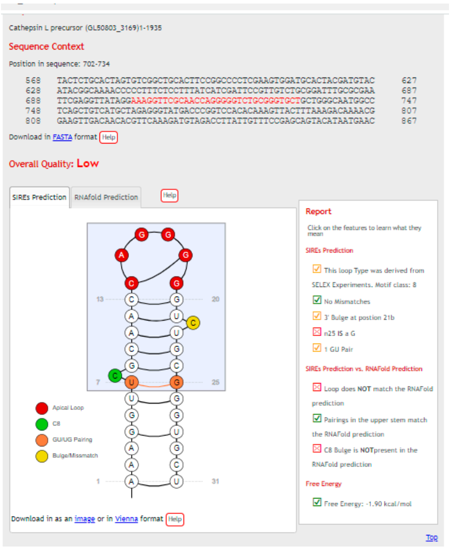

>Cathepsin L-like protease (GL50803_3099)1-1530
TGTAGTGTCTGATGAGGAACTACGTAAGCAGCGTGCAATACGACAGCGTGAAAAAACTGA
GCGGAAACAACGAAAATAGGAATTGTGTAAATAACGAAAATAATATTTAAAAGAACCATA
ATGTATACTTTGTTGGTGTTCATACTGTCGAATCCTCTGCATATCCTATTAAAAGCACGC
TCTTACACAGGAACCTTTTCGGCATGCACTAAGGACAATGCAGTTGTTCGTAGATTGCAA
TCTAACGATCTGTTTCTGCTGTTGGCTAATCCAATTAAAGTGGCCGCAGAGGACAACCTA
CCACAATCTGTTGACTTAAGGGAGTACGGCCTTATGACTCCTGTCAGAAACCAGGGTAAG
TGCGGCTCGTGCTGGGCGTTTGCAACCGTTGCAGCCTTCGAGGCGTGTGCCCTGTATGAT
GGCGTCCTTTACCCTTACGGAAGTCCACTTGGCAGACACACAGACCTCCTGCACATCTCT
GAGGAGTTTGAAATCTTGAGCTCCACGTCCAATAACCTCTGCTCAAGTGGCAACGTGGTG
CGATTGATTAATGACCACCTGACGGGAGTAATTCCCACTGTAGAATTGGAGGAAAACTTT
CCTTACAGTTTGAGTGACAATACTGAAAGCGAGCCCCCCGCACACCCCCCTGCAGCCTGCG

ATTCCACCCGAGGAGTATGTGCTTCCAATTTATTCTGAGACAAAGCTAGGGCATAAAGCA
GGGGTCATAGTGCTTTACAACAGAGACAATCCCTATAATGACTCAATTGCACAGCTGGTG
AAAACGTATCTGTCACTGGGGGTTCCTGTGATGGCTACGATTAACACGTGGGCTAATGGA
AGGAAAGGTCACCGGTTAATTAAGCGTTATCGCGGTGGCATTTTGAGCGCACCGTGCCGT
AATACAAGCCTGGATCACGAGGTGCTCATTGTTGGATATGGACGGTATCGAGGTACTGCC
GTTTGGATAGTTCGCAATTCTTGGGGAGCTGACTGGGGAGTCTATGGGTATGCATATGTG
GCCCAGGGCGGTGACAGCTACTGCTTGGAGCACAGTGCCATTGCTGTTCTTCCCCGGACA
CTCGATCCGACCGCCAAGTACGCATCCTTCTCTACGCATCCACCTCCGTCTCCGCTCCTC
CGGCCGATATATGAGGGCACTATTGTACGCTGTGAAAACGGGCTCGATGCTGATCCACCG
GAGGGTTGCAGCCCATTCACTAGGGGCAGCTCGCCCGCGCTCGGGGCCAGCATCGCCCTC
ATACTCACAACCATAGCAGCTATAGGGAAATTTACTCACCAGCCCAGCCCCTTAATCAAC
CTTCGTCGTCATGATCGCGTCGAAAATGCGAGTGTCAGGAACAAGGTCACTGAAGCAATA
ATCACCGAAGTGCTCGGAATTATCGGATAAGACAGTCATTATCTCGGCGAGATTCTTCTG
CATCATCATCTGGTTGAAGCGGCGCTCCTCCGAATTCTTCTTGAGGATGGGGAGATACTT
CGCCACGCTCTCTGCGAAGGACTTGATCTT

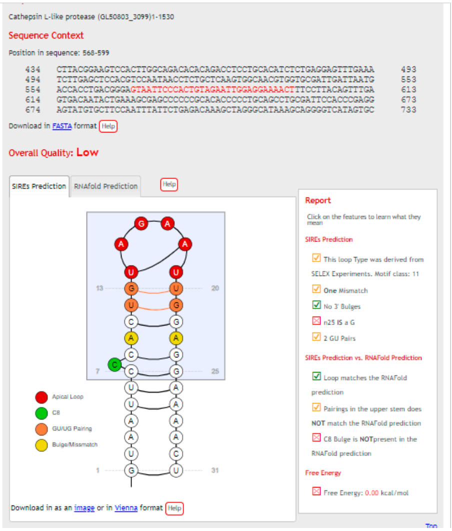

>Hypothetical protein (GL50803_113303)1-2655
CTGCAGAAGAGTAAAATGCAATGTAAATAGCCTCCTCAATTTTTATTCCACGGCTTTCC
CTGCGGGATTCCCTGCAGGTCTCCCTGCGCCACTGCTCCGGGAACGGCCACGGAAATAAA
ATGACGGTACTTCTACCATTCTTACTATTGCTATATGGCACACTTGGCAGAGAGCCTTCC
CCCTTGCGGGAGGGTGACGACCAGATGGACCCCGAGGGCGGCACGATCTTCCCTTCCGAG
GACGAGTACAACGAGCTTCCCGATGGACCCGACAACGCAGATCTGACCCGCGCAGCGCTC
CCTACCAACTTCACCTACCGGGGCCACCGGTGCATCCAGATCATAGACCAGGGCTCGTGC
GGCTGCTGCTACGCGGCGGCGGCGGTGGAGATGGTCACTGCCCGGCGGTGCCTCCAGCTC
AACGACAGCAGGCTTGTGAGCCTCGAGGACCTCGTCACGTGCGACCACACCAAGTATCTG
AACATCCAGAACAACGGGTGCCGCGGCGGCAATCCCCTTGCGTCGCTGAAGTTCGGAGAG
ACGACGGGCATGGTGTACGACACCTGCGAGGACTACTGGAACCGGACGTATCCCTACCCC
ACGGAGACGTGCAAGACCGTCTGCAAGGATAAGCGCCCCAAGGACCGCACCATTAAGAAC
AAGGCGCCCTACAGGCTCAGCGGCGTGGACGCGATGATGCGCGACATATACCAGAACGGG
CCCATTGCGGTCTCCATGTACCTGGCCAACGACTTCCCCTCCAAGGACAAGAAGGGCATC

TACAGCTCCGGCCCCAATACGAAGCTGGGGGGAGGCCACGCCGTGATGATAGTCGGGTGG
GGCGAGGAGAACGGCGTGCCCTACTGGGACTGTGCGAACACGTACGGCACCAATTGGGGC
GACCAAGGCTACTTCAAGATCAAGCGCGGGTCCAACGAGCTCAAGATCGAGACGTGGCCC
GGTTCAGCGCTGCCTATAGACACGACTCCTAAGCCAGAGCCCCGAAACGGCACAATCACT
GTAGAGACGGCCAAAACGTACATTGCCGGATACGAGGTTGCAATACCGTACGAGAAGGTA
GCGACCCCTGCAGAGCTCCAGCTGAAGTCAGGAGCAGGAGCGGTGACGTCGCTCGTTAAG
CTGACGACCTCTAGTGGCACCGCCCGCGTCACCATACCTCGAGACACGTCTGCAGGGCAA
TACTACCTGACAACCAGCACTGGAGACGCAACGTCTGCGCGCTTCTCGTTGGCGTCCTAC
TTCACGCTGGCATTCGACAAGGCTAGCTATCAGGGACAAGAGGGAGAGAGCGTCGCACTC
CGGTTCGCTGCACCGCTCCAGGTGCCCTGCAAGTTGATGGACGGCGCGTCTGTGCTGGTG
GAGCTCAGTAAGGGGGCGCAATCCTGCATGGCCCCGTCGAGCATGAGCGCTGGCAAGCAT
GCGCTGACCCTAACCACGTCCGACACCGTTCCCGTCCTATCCGCAAGCACAGTGCTCACA
ATAGAGAAGAAGCACAATGATGAAGCAGTCATCACGATATCTAAGCCGGTCGCCGGCGCA
CAGGTGACTGCGTTGGCCGAGGGCCTGCAGATTTCGTATACCTCAACGGGGACGGGCGCA
AAGCTGCTTCTCCAGGCGTATTGCGGAGAGCGACTGCTGGATGTCATCGCTGCTGGCCTG
CCTCCCTCGGGAAGCATCGAGGTCCGCCTGCCGTCTTCCTATGGCAACTGCTCAGCTGCG
TACCTCCGATTGCGCTCAGAGACGTCCCCCTTCGCGTACGCCGATGTGGGACCGCTGCAT
GTCACGTCGTACAGCTACCCTGCAGATAATCTCCCGGCCCCGTCTGATAAGAAGGATCCC
GTGCCCGACCCGGAACCACAGCCCAACCCAGATCCCGAACCTGAGCCCGAACCTGAGCCT
ATTGGCGACGTGCCCCTGCTTATTACGGAGCCGTCTACGCAGAGTGTCTGGGTCCCCGGC
GGTGCAGTTACAATCCGATGGACCAGTAACCTAACAGCCGGTACTGAGATGACGATCCTT
TTGTACGAAAAGGTCGGATCGAAAAGCTACCTGCGCCACACGTTCACCCGCTCTGCCCCT
AACACGGGGCTCTATGCCGACACTCTTCCGGCCTCTGTCCCCTCTGGCCCTAACTACTTC
GTCCGCATGCGCTCCAACAGTCCCATGTTCACGAACACTTCGGATGACTTTGAAGTCAAG
GCGAGCAGATACCTGGTCAGAGACGTTCCAGACGCCATTACTATGCGGGATCCTCTGAGC
TTCACTATCCATAGACACGGGTTTGTGTTTCCCTTGCCTCGCAGAAGGTTCTCGGTCACG
CTAATGCAGGCGGGAAAGGAGGTTTGTGCACTTGACGGAGTCGAGGGAGTGGCAGGACTC
AACACGATCTATCTAGATAGCTGTGCTGAGAGGATGCAGGAGCTCCGAGACAAACAATAC
TACCTAAAACTATGCTCTCAGAAAATGGAGTGCACGGTAACGCCGACTTTCACGGTATTG
GATGACACTCATTGACACACTCTGATGAACTAGATGGTAAATCTATCCTAAGCAAAGATG
TTGTCGGTTACACTGGACAGACTGTCATAAGCTCCTTCCCTTCATAGCCGACCGACTTTA
CCGAGGCTCCGAGAA

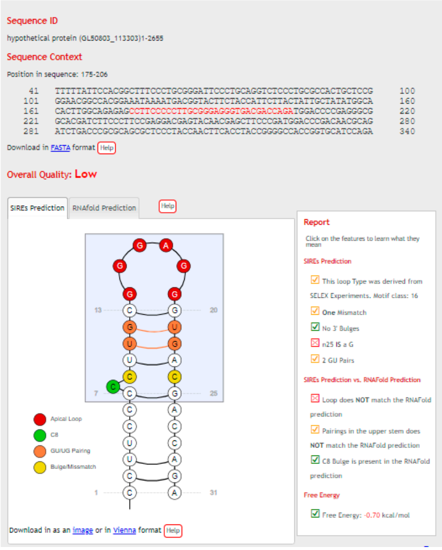

>Ribosomal protein L7a (GL50803_17244)1-1027
TAGTATGTTTAACACAATCGTAAACCGTTACTTCACCTAAATTCTATACCTGATCCCAGA
TTTAAAAAATAAAAACTAATTTAAATTTATTTCACTGGGTTCAATAAAACAGGCCTCGAC
ATGTCCAAGGTTTCTGGCAGCGACATTAAGAGGGCCCTCGCCGTACCCGAGAACAAGAGC

CGCAGCAAGTGCGACTTCGACCTGACTCCGTTCGTCAGGTGGCCTCGCCAGGTCCGCATC
CAGAGACAGAAGGCAGTCCTCCAGAGGCGCCTCAAGGTTCCCCCAACTGTCAATCAGTTC
ATGAATCCGATCTCGAGGAACCTCACAAACGAGATTTTCAACCTTGCTCGCAAGTACTCC
CCCGAATCGAAGGAGGAGCACAAGGCGCGCCTGCTCCAGATCGCCGACGCAAAGGCCAAC
GGGAAGCCCCTTCCGGAGAAGTCTGACAAGCTTGTCATCGCGTCTGGTATCAGACGCATA
ACATCCCTCGTCGAGAGCAAGCGCGCGAAGCTTGTCCTGATTGCAAATGATGTCGACCCC
CTTGAAGTATGTTCTTATGCGCGAGGAGCCGTCCGCTGACCGCACACACCTCTGATTGCG
GGTTGTGTGTTGTCAGCGGGTGGACTTCGCTGTTCACCTGACAACTGACCCACAGCTCGT
ACTTTGGCTTCCCACACTCTGTCACAAGATGGGCGTCCCGTACGCCATCGTTCGCACTAA
GGGCGATCTGGGCAAGCTCGTCCATCTGAAGAAGACGACCAGCGTCTGCTTCACCGACGT
GAACCCAGAGGACAAGCCCACCTTTGATAAGATCCTCGCGGCCGTGGCCCATGAAGTTGA
TTATGCAAAGGCCATGAAGACGTACGGAGGCGGCGTTCGCCGCGAGGATGAGGCCCAGCA
GATGTAAATGCCTTACATTTCTTTAGTGCATGTCCTTTGCTGCTTGGTCCTTTGTGCTCG
GGCTGTGCACCTTGGTAGGCCTGGTCTTGTTTCGCCGTCCGCAGCGCAAGCCCAGTGACA
CGACGCC

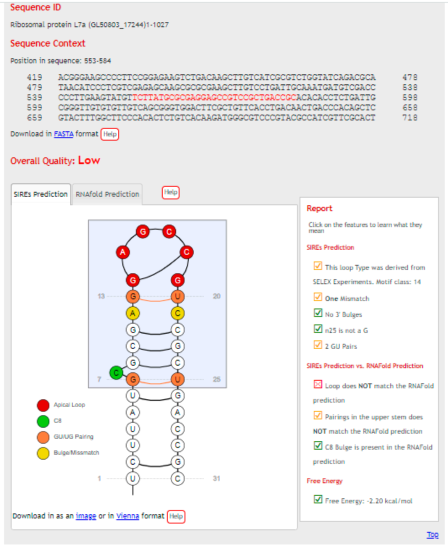

>Ribosomal protein L12 (GL50803_14938)1-789

GTGGGGAGTATGCCCAGACACTTTTTACGTCTTTATTTCATTTGTCCAATTAATAATATA
AATTATAGTTAAAGCATTTTAATTTGATATTATGTCAGAGTGGTAGTTTTCCAGGCGACA
ATGCCCCCCAGAGCTGATCCTAACGCAGAGATAGTTCTGTATTTGCGCGTCAAGGGCGGG
ATAATCCCCGCGCCAAATGCCCTTGGTCCAAAGATCGGGCCCTACGGTCTTCCGCCCAAA
GTTGTTGGTGAGAAGATCCACGAGGCTACACAAGGCTACAAGGGGATCCGCGTGAAAGTC
CGCATCGTCTCAAAGAACAGACAGCCGACAGTCTCTGTTGTGCCCACAGCCTCCTCGCTT
CTTGTCAAGGCTCTGGGCGAGGGTCCTCGCACGATTCCCAAGGGTCAGCCCCTTCTGCAC
ACAGGCACAGTGAAGTTCGACACGGTCCTAGACATCGCAAAGGAGCTCCGCGCTAACTCC
TTCGCTCTGAAGTACGCCGGCACGGTCCTCGAGGTCCTGGGTTCTGCCCGCTCCGTTGGC
TGCAAGGTGGAGTACAAGGGCACTGTATATTCTCCCGCTGAGATCACGGAGATGGTGAAG
GCCGGGGACATAGAGATCCCAGAATATAATGTACCCTACCAGCTTGCTGAGGAGCAAGAG
ACAGAGTAAACAAGGTGCCCTGCGTGTCACGGATCGGTCCCTATCTGCTTGGGACTGTTT
TCGCCTACGGGGGCTTAAAATATTTCGATGGCCGAGGCGGCGGAGTCCTTTGCCGATGCA
GTCGTCCGT

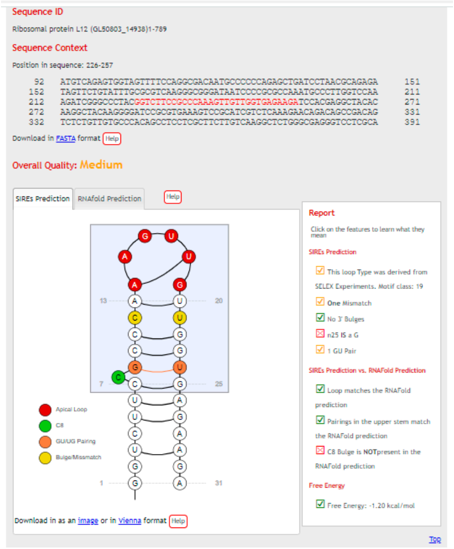

>Actin related protein (GL50803_8726)1-3147
CCTGCTCTAAATTTCATCCAAATTTCATTTTATAAATAAATTCTGCGACAAGTTAATTTA
AAAGAAAAGGACATATTCTTTGGAAAGCTTTTGTATTTTGGAATTTGACTGAAATTATAA
ATGTCTAGACCCCTGACCTCTCTTCGTGCACACTCCGGAAGCACCTTTCAGCATTCGGCG
GACTTGCGTAACGAGCTCGAAGAGGCCATTCTCGCATATACACATGGATACGCCGACAAT
CTTCGCCAGTTTTTCTTGCAGAATGATAAGGCCAAGACGGGGCAGCTCTCTGACATTCTG
TTCCGCCGGGCGCTTCGCTCCATTGGTATCCACGGACCAGTTGAGCTGCTTAATCGTGCA
ATAGAAGAGGCCACTGACCTTAAGAGTGGACTAATAGATTATGAGGCGTTTTTGGGGCTC
TTTGCGTTTAAGGGCGACATACCCCCCGAGGCTGAACGAGGAACATCAACCATTGTTCAT
AGCCGATACGTGGGTAATTACGCAGAGCTTGACTCTCAGAAAAGCAAGAGACCTTCCTCG
CGTTCAGCCACAAATCAGAGACAGCATCATGACGAACCCCTCAACCTTGCTGATGTTGAA
GGTGATCCTCTACCTCAGGAGGAAGTGATTGCCGGCGCACCCTCGAGCAGAAGCACGTTT
AATCTGCAGGAGGAGATGCGCAAGGCAGCGGATGACTTAAAGCGCATGCAATCCGAAACA
AATACAGAATTTCAAGTTGGATTGAAGCTCATTGCGACCCAAGTACTTCAAAAGGGCTCT
TGTGTTAAGGTTTTCCGAACGAAATTTGACAGAAACCACAAGGGAAATATAGGGGTCCAA
GACTTCCTTCTGGGTGTTCGATACCTAGGGATACCATCTGATCTGATTAGCAATGATGAT
CTCGTCGAAATTTTCCAATCGTACTGCACAGAGGACGGCTCCATCTCGGTCATTGAGTTT
GAGGCTATGGTGAACGCCGGTGAAGACACCAAGGACGTTACCCGGACTATGCACGATCGT
CAAGCAAGGTCCACCAGAGACCCGACCCATGATGTGGCTCTTCTGCTGGAGGAAGCCCAG
ACTGTTAGTGCAAGTGGGATCTCAGGTGAAAGGAAACCCTTACGTCGCACTCAGGGCACT
CCAGGAAGGAATCTAACTGTTTCCAATCTTGCCACAAACCAGGGATCTGATCCTGCTTGC
TCCTCAGCGCCTAGTGTTGCTGCAAGCACAACTAACCGACCATCTAAGTATAGTGATGAG
GAAATAGTAGGAACGCTCAAGCGAAAAATTGCATTGATATGCTCTAACAAATACTCCTCT
TTTGGTGATTGCATGGCTGATGCCCAAGACTACAACAGAAAAATTACACCAGACTCGCTG
CGCAGGTTTGTTAGCTCTCAGGTGCCAAATTTAGATAAGAGATACGTCAATCTACTCGTC
TCATACCTGTGCACACAGGAGAATCTCAGACAGAGTGCAAATTCGCTTGGGATAAGCATA
GCGGAAATACAGCAGAGCCAGGTGGGAGCATCTAGCATGCCGGAACCAGCCTACATATCG
GCAGTAGTTTTCAATGACCTTTACAAGCTTCCTACCGCTACAGACGGCGGGAAGAGACGC
ATAGATGATGCTATCTGGACATGCATCACAACAGAGGCCAATCTGAGTAACATGGCTCTT
TCTAAGGCAGAGCTTATGGCCATATCTGTTAACAACCAGAGGTCGCAGGGGCTATACACT

GATGACCCCCCTACGCCCTGTGAGCTCCAGACCCATGCTTCGGTGTTTGGTAACCTGAAC
ATGGCTGAGGGCCGCCAGGGTGGGTCTCAGGGTGCCGCCATGGCCCAGGAGATCGCGCAG
CATCAGCGCCACACTAAGTCTGGAAATTACTTTGATACCACCGCGGCCCAAAATGATGAC
ATCACGTCCTACGTCAACTGCAGTAATGTCAATATTGCGAACCCCGACACCCCCTCTACT
CGCCGTCTCAGACAGTCTGGTCGTCAAGCCGAGCTTCTAACAAGCCAGCTCCAGAACCCC
GCCTCGTCTACTCAGGCAGAGCGCGCCAGCGAGATAATACGTACTAAAAGACAGCTCCTT
GAGGCAATGAACAATAAGCGGAGCGCACTGGCAGAGGCCTTAATCATGGCAGCAGATCGA
GCGACAGATCCAATATCATTCAGAAACTATCTCAAGGACTTTACTCAGGAGTCTCTGACT
AATAACCAAATACAATGCATTCTTGAGGGCTCTCTGGATTCTAACGGCAAGATCCAAAAG
GAGATCATACTCAAGACTATGGGCCTCGATAACGAGTTTATAGCATATGCAGATTCCGGT
TTGGAGAGTTCGAAGCTGAAGAACGATCGTGTTTCTGACAGTATGGTGTGCATCTCGGCC
GTTCGCGAGCAAATGAATGCTTCGATAAGCGGAGCGCCACGCGATATGATCTCATCCAAG
CGTGCCATGCAGGCCAGACAAAAGCAGGAGTCATCTATAATGAGGCAGCTGTCGTCTGCA
GACCTAGATCTTATCAAACCTCGTACTCCAAACATGAGCACAGTGGCAAAGCATCAGGAT
AGCTCTGCACTCGCCAAGGTTGTATTCGACGTAGAGAAGAATATCACTGAGGATTATGAG
GGCTGCCTAGCAGAAGCTACCGAGGCCATTGTTGGGGGACACAGAAGGCGCCACCTTGGC
GGTGACCTCAATAAGAAGGACAACTTGGTTATGAGCAATGGAGGAGTATACTCACTGCGA
GACAGACAACTAGAAGAAGCAGAAGCAAACACCCGACGAATCAAGAACACCCAAAGGTCT
GTCATGGAGCACTCCACCCCCGCCGATGCTATTCCACGACGTGGACGCGGGTGCAATCAG
GCTGTATCGCAGCTAAGCAGCACAATTGGTAACACACTTAAACAAATTGATGAAGAATAT
CTTGTAGATTATCAATCTGGCCAAATTGATGAAACTGCCCCTGCTGAAGGAGGAAGTGTC
CCGAAACCAGTTGCCTTTGAGGTTTGAAAGGCCTCGTGTCTATGGTATATACTATTTCCG
ATGAATAAATCGCTTTGTTTTTGTCATGCATGAAAATTTGACTTAAAATTTCGCCCTGTG
ATTTGCGAGGTCTATGAAACAGGCTGC

Sequence ID

Actin related protein (GL50803_8726)1-3147

Sequence Context

Position in sequence: 1511-1543

```
1377   GCTGCGCAGGTTTGTTAGCTCTCAGGTGCCAAATTTAGATAAGAGATACGTCAATCTACT   1436
1437   CGTCTCATACCTGTGCACACAGGAGAATCTCAGACAGAGTGCAAATTCGCTTGGGATAAG   1496
1497   CATAGCGGAAATACAGCAGAGCCAGGTGGGAGCATCTAGCATGCCGGAACCAGCCTACAT   1556
1557   ATCGGCAGTAGTTTTCAATGACCTTTACAAGCTTCCTACCGCTACAGACGGCGGGAAGAG   1616
1617   ACGCATAGATGATGCTATCTGGACATGCATCACAACAGAGGCCAATCTGAGTAACATGGC   1676
```

Download in FASTA format Help

Overall Quality: Low

SIREs Prediction | RNAfold Prediction | Help

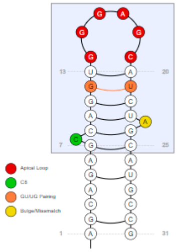

Download in as an image or in Vienna format Help

Report

Click on the features to learn what they mean

SIREs Prediction

- This loop Type was derived from SELEX Experiments. Motif class: 16
- No Mismatches
- 3' Bulge at postion 23b
- n25 is not a G
- 1 GU Pair

SIREs Prediction vs. RNAFold Prediction

- Loop does **NOT** match the RNAFold prediction
- Pairings in the upper stem does **NOT** match the RNAFold prediction
- C8 Bulge is **NOT**present in the RNAFold prediction

Free Energy

- Free Energy: -3.00 kcal/mol
